# Reinforcement learning for LLM-based explainable TCM prescription recommendation with implicit preferences from small language models

**DOI:** 10.1186/s13020-025-01250-7

**Published:** 2025-11-19

**Authors:** Xinyu Wang, Xiaohe Sun, Lei Yang, Yitong Zhang, Tao Yang, Jiadong Xie, Kongfa Hu

**Affiliations:** 1 School of Artificial Intelligence and Information Technology, Nanjing University of Chinese Medicine, Nanjing, 210023 China; 2 Jiangsu Province Engineering Research Center of TCM Intelligence Health Service, Nanjing University of Chinese Medicine, Nanjing, 210023 China; 3 Tang Zhongying Research Center for Traditional Chinese Medicine Epidemic Diseases, Nanjing University of Chinese Medicine, Nanjing, 210023 China; 4 The First Clinical Medical College, Nanjing University of Chinese Medicine, Nanjing, 210023 China

**Keywords:** TCM prescription recommendation, Knowledge distillation, Direct preference optimization, Implicit preference, BART

## Abstract

**Objective:**

In an effort to reinforce both the interpretability and accuracy of prescription recommendations in Traditional Chinese Medicine (TCM), this study puts forward a two-stage training framework that integrates knowledge distillation from a teacher model with implicit preference-driven reinforcement learning grounded in a compact model.

**Methods:**

Above all, GPT-4o is employed to parse structured TCM clinical records, creating high-quality distillation samples. These are employed to guide Low-Rank Adaptation (LoRA)-based fine-tuning of the Qwen2.5-7B model, enabling it to generate explainable outputs in the format of "symptom analysis—prescription recommendation—prescription explanation". Then, a lightweight BART (Bidirectional and Auto-Regressive Transformers) model is trained to learn the mapping relation between symptoms and prescriptions. Its outputs are compared with those of the large model to construct preference pairs, which are subsequently utilized in Direct Preference Optimization (DPO)-based reinforcement tuning to further align the model with potentially better recommendations.

**Results:**

The suggested model achieves a *P*@30 of 35.62% and *F1*@30 of 37.36%, outperforming existing baselines. Knowledge distillation contributes to the improvement of the model's generalization and explainability, while implicit preference-based reinforcement further enhances *F1*@30 by 2.01%. Overall, the model obtains more desirable performance in both accuracy and explainability.

**Conclusion:**

The recommended approach not only improves the quality and transparency of TCM prescription recommendations, but also offers a fruitful strategy for building trustworthy and clinically applicable intelligent TCM decision-support systems.

"Pattern differentiation and treatment on the basis of syndrome variation" lies at the heart of traditional Chinese medicine (TCM), emphasizing individualized prescriptions and therapeutic adjustments in alignment with the distinct syndromes exhibited by the patient throughout various phases of disease advancement [1]. Over years of clinical practice, TCM has accumulated numerous medical case records, encompassing rich information such as the Four Diagnostic Methods, syndrome analysis, and herbal prescriptions, thereby providing valuable resources for the modernization of TCM research [2]. Nonetheless, owing to the highly abstract nature of TCM theory and the individualized nature of its treatment strategies, it remains one of the pivotal challenges to extract prescription recommendation logic that is both explainable and aligned with TCM principles from large-scale, and heterogeneous clinical case data, especially when it comes to how we can accelerate the intelligent and standardized application of TCM in clinical practice.

As a multitude of extensively acknowledged models such as GPT-4o [3], DeepSeek-R1 [4], LLaMA3.1 [5], and Qwen2.5 [6] have been released successively, large language models (LLMs) have achieved remarkable performance not only in natural language understanding and generation tasks, but also in domain-specific adaptation. These models have obtained impressive performance and demonstrated conspicuous domain adaptability in language understanding and generation tasks. Through multiple techniques such as pre-training [7], supervised fine-tuning [8], reinforcement learning [9], and few-shot learning [10], they can be effectively transferred to specialized domains encompassing healthcare [11], education [12], and law [13]. Thanks to their exceptional context modeling [14] and Chain-of-Thought (CoT) reasoning capabilities [15], LLMs have demonstrated promising potential in TCM tasks such as knowledge-based question answering, diagnostic reasoning, and prescription recommendation.

Given this context, investigators have enthusiastically probed into pathways for integrating LLMs with TCM, offering a range of tailored models, which are exclusively designed to meet the demands of TCM tasks. For instance, Lindan [16] developed a TCM-specific large language model by integrating multi-source data comprising classical texts, textbooks, and clinical records, and incorporating CoT reasoning to support diagnosis, treatment, and prescription recommendation. TCMLLM-PR [17] constructed a dataset of over 60,000 TCM instruction samples and applied P-Tuning v2 on ChatGLM-6B, which eventually materialized F1@10 reinforcements of 31.8% and 59.5% on textbook-based and pharmacopoeia-based prescription tasks, respectively, with outputs approaching real prescriptions. JingFang [18] innovatively incorporated a Multi-Agent Collaborative Chain-of-Thought Mechanism (MACCTM) and a Dual-Stage Recovery Scheme (DSRS) to strengthen the model's performance in clinical inquiry and syndrome differentiation. ShenNong-TCM was trained on over 110,000 TCM-related instructions generated via a self-instruct strategy centered on medical entities and leveraging ChatGPT, ultimately reinforcing its properties and enhancements on TCM knowledge-based Q&A tasks. HuatuoGPT-o1 [19] incorporated verifiable medical prompts and a medical validator to guide complex reasoning fine-tuning and reinforcement learning optimization, surpassing multiple baseline models with only 40,000 training samples and strengthening the model's ability in clinical problem-solving. Nevertheless, current TCM LLMs continue to confront a range of significant constraints: (1) the integration of structured knowledge with interpretable reasoning is hindered by fundamental flaws, which eventually renders it considerably challenging for models to generate prescriptions accompanied by a complete and traceable chain of logic, thereby constraining the credibility of recommendations. Existing studies have systematically put forward explainable AutoML approaches in the medical domain [20], yet these approaches entail more systematic investigation in the context of TCM LLMs. (2) supervised training objectives are singular, within which models are centered around nothing more than fitting existing data and lacking explicit guidance toward generating "high-quality prescriptions", which limits their ability to optimize treatment strategies proactively; and (3) reinforcement learning strategies lack clearly defined and stable reward mechanisms for TCM diagnostic tasks, thereby giving rise to unreliable improvements in model-based clinical performance.

In an effort to mitigate these issues, this study comes up with a two-stage training framework that integrates supervised fine-tuning via knowledge distillation with implicit preference-driven reinforcement learning rooted in a compact model. To be specific, (1) GPT-4o is adopted as the teacher model to delve into structured TCM case records and generate high-quality demonstration samples incorporating a three-step logic, "symptom analysis—prescription recommendation—prescription explanation", thereby constructing a training set with coherent reasoning chains. LoRA [21] is subsequently employed to fine-tune the Qwen2.5-7B model, enabling it to perform explainable prescription recommendation. (2) A lightweight BART model [22] is trained on symptom–prescription pairs from clinical case records to serve as a reference generator. Symptom data is further reinforced to reinforce alignment diversity. Preference data is constructed by comparing BART-generated outputs with Qwen2.5-7B outputs under different decoding temperatures. Finally, Direct Preference DPO [23] is employed to perform reinforcement fine-tuning, aligning the model outputs toward more desirable prescription candidates. The principal contributions of this study are outlined as follows.

- ① A knowledge distillation-driven strategy for TCM prescription recommendation is recommended: By guiding the teacher model to generate training samples with both symptom analysis and prescription interpretation, the student model is encouraged to generate logically structured outputs, thereby strengthening the interpretability of recommendation results.
- ② Application of DPO to TCM prescription tasks is introduced: It is to introduce DPO into TCM modeling, tremendously simplifying the reinforcement learning process while ensuring the training runs smoothly and effectively in comparison with traditional reinforcement learning.
- ③ Implicit preferences from a compact model are utilized to guide LLM training: By employing the output of a compact pre-trained BART model as a proxy for implicit preferences, this approach mitigates the data bottleneck resulted from the lack of large-scale human-labeled preference data in the TCM domain, while also reinforcing the accuracy and reliability of LLMs in prescription recommendation tasks.

## Methods

**Fig. 1:**
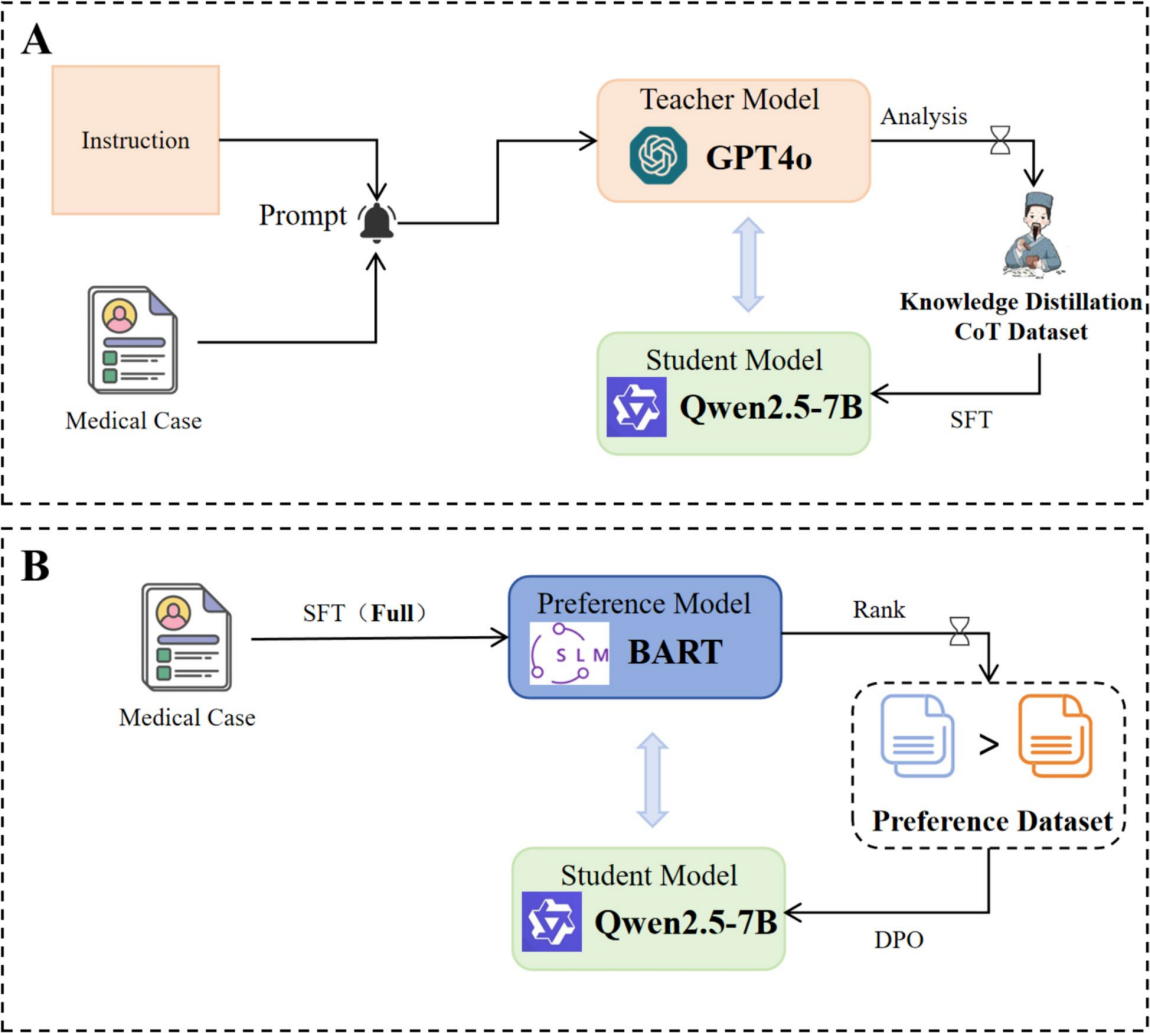
Overview of model construction and training workflow

This section delves into the construction of the suggested model, which consists of two primary components: (1) CoT-based supervised data generation via knowledge distillation and supervised fine-tuning; and (2) implicit preference modeling by adopting a BART model and reinforcement optimization via DPO. The overall workflow is illustrated in Fig. 1. To start with, we design a structured instruction template to guide the GPT-4o model in standardizing and parsing TCM case records. Outputs are generated in a three-part format comprising "symptom analysis—prescription recommendation—prescription explanation". These high-quality samples serve as supervision data to fine-tune the Qwen2.5-7B model via the LoRA, facilitating the model's generation of TCM prescription recommendations that are interpretable and explainable. In particular, the model not only learns to generate herbal prescriptions but also can provide corresponding syndrome differentiation and formulation rationale. Afterwards, a compact BART model is trained to learn the symptom-to-prescription mapping. After the above steps, the symptom descriptions in the training set are ameliorated to enrich the alignment data, and the BART model is employed to generate accurate reference prescriptions. These references serve as a proxy for implicit preferences. Preference pairs are formulated by assessing the similarity between the outputs from the large model and those prescriptions produced by BART. The model is subsequently further fine-tuned by adopting DPO, aligning its outputs with implicit preferences to enhance its domain alignment, output stability, and explainability in prescription recommendation. Ultimately, this two-stage methodology dramatically helps establish a trustworthy TCM large language model.

### CoT-based supervised data generation via knowledge distillation and supervised fine-tuning

In an effort to strengthen the adaptability of large language models for explainable TCM prescription recommendation tasks, this study adopts a two-fold strategy combining knowledge distillation [24] with supervised fine-tuning for initial optimization.

**Fig. 2:**
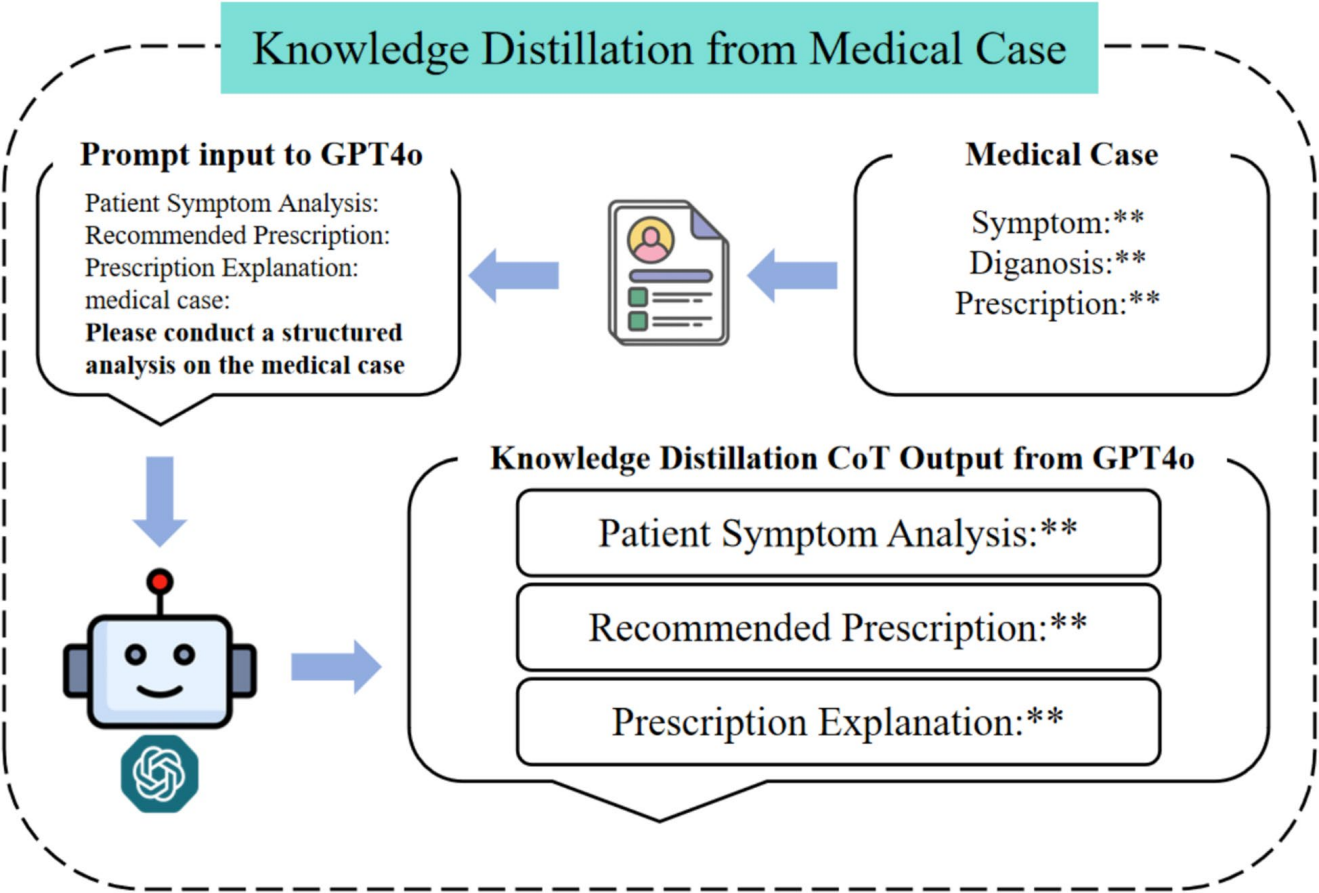
Instruction template for output generation

GPT-4o is selected as the teacher model to perform structured parsing of TCM medical records, thereby constructing a high-quality supervised training dataset that reflects a CoT reasoning process. Guided by the TCM principle of "syndrome differentiation and treatment", we have designed a three-stage output format that encompasses symptom analysis, prescription recommendation, and prescription explanation. Through a few-shot prompting approach, in which representative exemplars are provided, GPT-4o is prompted to generate CoT-based supervised data that embeds logical reasoning supported by TCM theoretical underpinnings. The prompt is summarized below: It is advisable to strictly follow the case-based format and provide the underlying reasoning process, including three components namely, patient symptom analysis (with TCM diagnosis derived from the symptoms), recommended prescription (list prescription only without specifying therapeutic effects, with prescription names unchanged), and prescription explanation. To ensure adequate scale, coverage, and diversity, CoT samples were generated for all training cases, thereby achieving full dataset coverage rather than partial sampling. While the output format was constrained for consistency, content diversity naturally arose from the heterogeneity of the underlying clinical records, which encompassed multiple disease categories, syndrome patterns, and prescription styles. Throughout this process, we meticulously preserved the clinical authenticity of the original case records while ensuring that the outputs possessed a structured, learnable format and remained readily explainable. The instruction template is demonstrated in Fig. 2.

For the outputs of knowledge distillation, this study employed an expert review mechanism. In detail, professionals with clinical backgrounds in TCM were invited to systematically assess the quality of the teacher model's outputs, scrutinizing syndrome differentiation logic and the appropriateness of herbal efficacy matching on a case-by-case basis. Subsequently, instances with evident errors were removed and re-analyzed until satisfactory results were attained, thus guaranteeing the precision of the training dataset.

When it comes to the selection of the student model, this study opts for Qwen2.5-7B. This model is constructed based on a decoder-only Transformer architecture and exhibits remarkable performance in Chinese language understanding and generation tasks. The model is pre-trained on a multi-lingual corpus comprising up to 18 trillion tokens, thereby rendering it tremendously suitable for processing text data in the TCM domain. In comparison with its predecessor Qwen2, Qwen2.5 displays heightened superiority in general knowledge reasoning, complex inference, and structured output generation. Paramount achievements in terms of architectural optimization are summarized below: (1) the use of Rotary Position Embedding (RoPE), which encodes positional information directly into the query (Q) and key (K) matrices via rotation, enabling the model to capture relative positional dependencies naturally in self-attention; (2) the integration of the SwiGLU activation function [25], combining gating mechanisms with linear transformations for more expressive representations; (3) the replacement of LayerNorm with RMSNorm to improve training stability and convergence speed; and (4) the introduction of Attention QKV biasing to enhance the model's representational and learning capacity. By striking a balance between performance and computational cost, Qwen2.5-7B is thus selected as the foundation for local adaptation.

Supervised fine-tuning of the Qwen2.5-7B model is conducted by employing LoRA. Moreover, LoRA enables effective domain adaptation with minimal parameter updates by injecting trainable low-rank matrices into selected layers. This approach dramatically reduces memory overhead and the risk of overfitting, while allowing the student model to effectively internalize the diagnostic reasoning and clinical knowledge distilled from GPT-4o. For this reason, the model's performance is enhanced across structured output tasks such as symptom analysis, prescription recommendation, and prescription explanation.

### Implicit preference modeling by adopting a BART model and reinforcement optimization via DPO

**Fig. 3:**
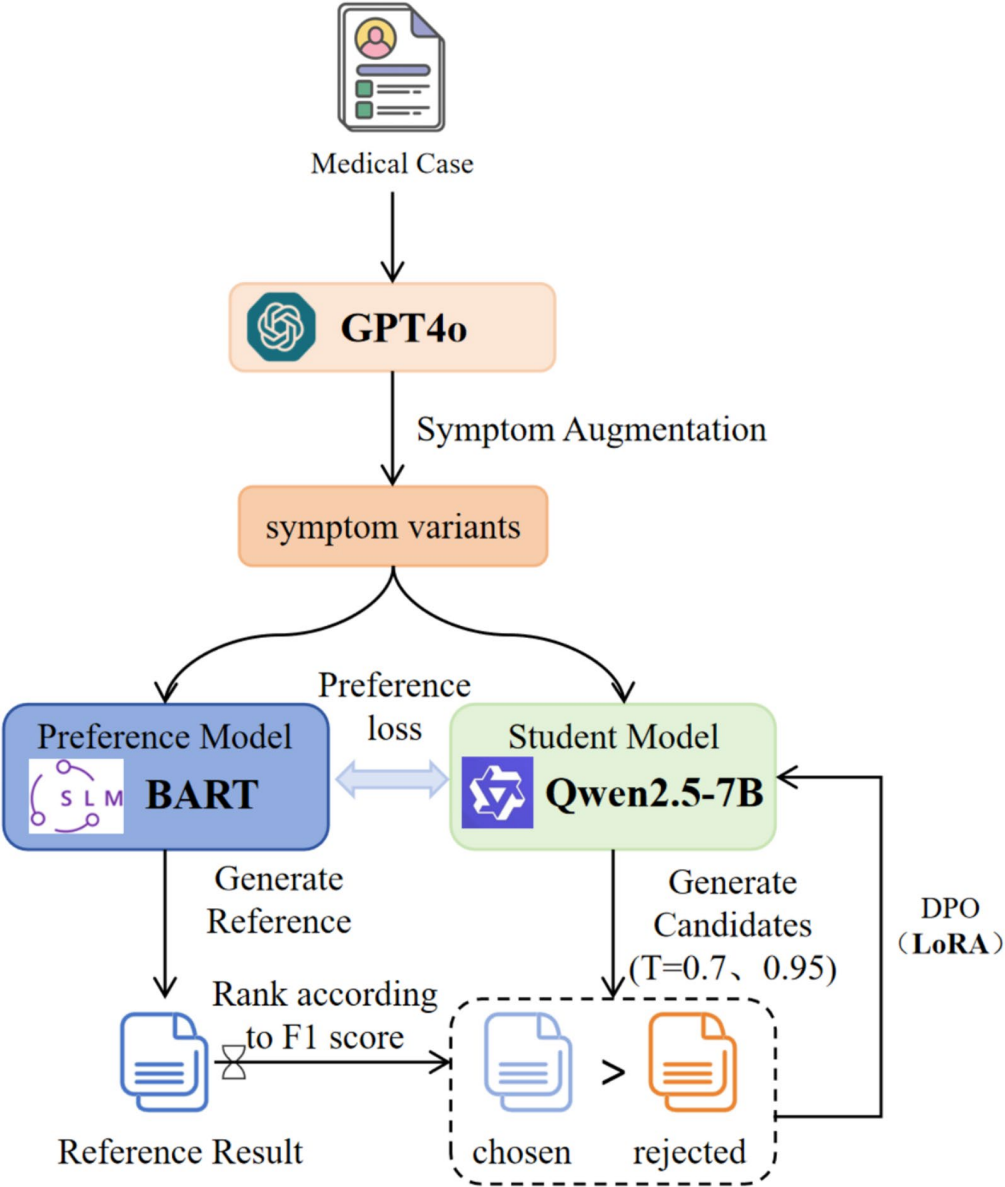
Framework of Preference-Guided Reinforcement Tuning

In the supervised fine-tuning of LLMs, the primary goal of typical training is the next-token prediction rooted in previous context, optimized via Maximum Likelihood Estimation (MLE). Despite the fact that this approach improves the fluency of generated text and its alignment with training data, it may bring about substantial output deviation, thus rendering it particularly demanding to guarantee optimal performance with regard to domain-specific accuracy and user preferences, particularly in tasks such as TCM prescription recommendation. As a consequence, strengthening recommendation quality is dependent on the effective construction of preference data. Nevertheless, conventional reinforcement learning methods frequently hinge on extensive manual preference annotation, a process that is both financially burdensome and practically challenging within the context of TCM. To address this limitation, this study introduces a relatively lightweight BART model, which is fully fine-tuned on the "symptom-to-prescription" mapping task to achieve more precise learning. The output of this model serves as a source of implicit preferences, which are subsequently utilized to guide the reinforcement tuning of the larger model. This approach mitigates the scarcity of labeled preference data and improves the model's recommendation quality. The overall process is illustrated in Fig. 3.

By adopting a structured instruction-based prompt (Please augment the patient symptom description '" + text + "' to guarantee superior diversity. Output four variants, one per line, without numbering.), patient symptom descriptions from the training set are first expanded via GPT-4o to construct the preference dataset, thereby generating four semantically diverse variants for each original symptom entry. These diversified inputs are subsequently fed into the knowledge distillation model to generate candidate prescriptions under various temperature settings. The candidate outputs are compared with the reference prescriptions generated by the lightweight BART model for the same inputs. By computing the similarity between each candidate and the reference by utilizing the F1 score, the system identifies chosen-rejected output pairs grounded in relative quality. These are subsequently utilized to build a preference dataset in the format of "Instruction—Input—(chosen, rejected)".

The BART model, developed by Meta AI, is a Seq2Seq Transformer architecture that integrates the text comprehension capabilities of BERT [26] encoder-only with the generative strengths of GPT [27] decoder-only. It is pre-trained via a denoising auto-encoder and is perfectly suitable for paired input–output modeling tasks. In contrast to large-scale LLMs with tens or hundreds of billions of parameters, BART offers a more compact architecture (BART-Large has 406 M parameters approximately), thus rendering it computationally efficient and particularly suitable for fine-tuning on smaller datasets. This feature enables it to effectively learn semantic information embedded in medical case records. As illustrated in Table 5, the BART model presents superior learning capacity in prescription recommendation tasks and serves as a reliable "auxiliary expert" for providing implicit preferences in guiding the optimization of large models.

On the basis of the constructed preference dataset, the DPO methodology has been adopted to perform reinforcement fine-tuning on the Qwen2.5-7B model. Unlike conventional reinforcement learning approaches such as PPO, it is unnecessary for DPO to require training an additional reward model or engaging in complex policy sampling. In contrast, it directly optimizes model parameters rooted in preference pairs, thereby simplifying the training pipeline while enhancing its stability.

The core idea of DPO is to adjust model parameter $\uptheta .\text{ In}$ such case, given a fixed input $x$, the probability of generating the preferred response ${y}_{chosed}$ is increased as a consequence of the less preferred response ${y}_{rejected}$. The corresponding loss function is defined as:

$${\mathcal{L}}_{DPO}\left({\pi }_{\theta };{\pi }_{ref}\right)=-{\mathbb{E}}_{\left(x,{y}_{chosed},{y}_{rejected}\right)\sim \mathcal{D}}\left[log\sigma \left(\beta log\frac{{\pi }_{\theta }\left({y}_{chosed}\mid x\right)}{{\pi }_{ref}\left({y}_{chosed}\mid x\right)}-\beta log\frac{{\pi }_{\theta }\left({y}_{rejected}\mid x\right)}{ {\pi }_{ref}\left({y}_{rejected}\mid x\right)}\right)\right]$$

Here, ${y}_{chosed}$ denotes the preferred response selected from the preference data, while ${y}_{rejected}$ defines the less preferred response. ${\pi }_{\theta }\left({y}_{chosed}\mid x\right)$ symbolizes the input $x$, and $\upbeta$ refers to the scaling coefficient of preference strength. ${\uppi }_{\text{ref}}\left({\text{y}}_{\text{c}h\text{osed}}\mid \text{x}\right)$ denotes the probability that the current function generates the preferred response given the input $x$ Through this two-stage training framework, the resulting model achieves both reliability and accuracy in the TCM prescription recommendation task. For one thing, knowledge distillation and supervised fine-tuning endow the model with coherent diagnostic reasoning and prescription interpretation capabilities, dramatically elevating the explainability of its outputs. For another, the refined model exhibits significantly improved alignment with real-world clinical prescribing patterns by integrating implicit preferences distilled from a smaller-scale model and implementing reinforcement tuning through DPO. This contributes to a more significant enhancement in the stability and precision of prescription recommendations, thereby establishing a robust technical foundation for building trustworthy intelligent TCM recommendation systems.

## Experimental settings

### Experimental setup

In this section, all experiments were conducted under consistent conditions to eliminate the influence of environmental and configuration variability on model performance. The hardware setup includes an Intel(R) Xeon(R) Gold 6430 CPU and four NVIDIA L20 GPUs (48 GB each). The software environment consists of CUDA 12.8, Python 3.12, PyTorch 2.5.1, Transformers 4.23.0 and llamafactory 0.9.2.dev0. The Qwen2.5-7B model was fine-tuned by utilizing the LoRA approach with a learning rate of 0.00005, employing a cosine decay scheduler for gradient optimization. In the LoRA configuration, the rank was set to 8 and the scaling factor (alpha) to 16. The number of trainable layers was restricted to two, with low-rank adaptation modules injected into the final two hidden layers of the model. LoRA was applied to all target modules (i.e., q_proj, v_proj, and o_proj). No dropout was applied during training, AdamW was selected as the optimizer, warmup steps were skipped, gradient accumulation steps were set to 8, and the maximum sequence length was 2,048. Training was conducted with bf16 precision, excluding quantization or gradient clipping. Throughout the knowledge distillation phase, the training process was designed to empower the model to fully capture semantic relationships within the TCM diagnostic corpus. In particular, training was conducted for 50 epochs with a batch size of 6. With an aim to prevent the model from converging to less desirable local minima throughout the reinforcement learning stage, the BART model was trained for 50 epochs with a batch size of 4, while the Qwen2.5-7B model was fine-tuned for 1 epoch with a batch size of 2. The DPO β was set to 0.50. During inference, the decoding parameters were configured as follows: temperature = 0.95, top-p = 0.70, beam size = 1, and repetition penalty = 1.0. For preference data collection, decoding temperatures of 0.70 and 0.95 were employed to generate diverse outputs, resulting in a total of 11,664 preference pairs. Every other hyperparameter was maintained at a constant setting, with no alterations made.

### Datasets

**Fig. 4:**
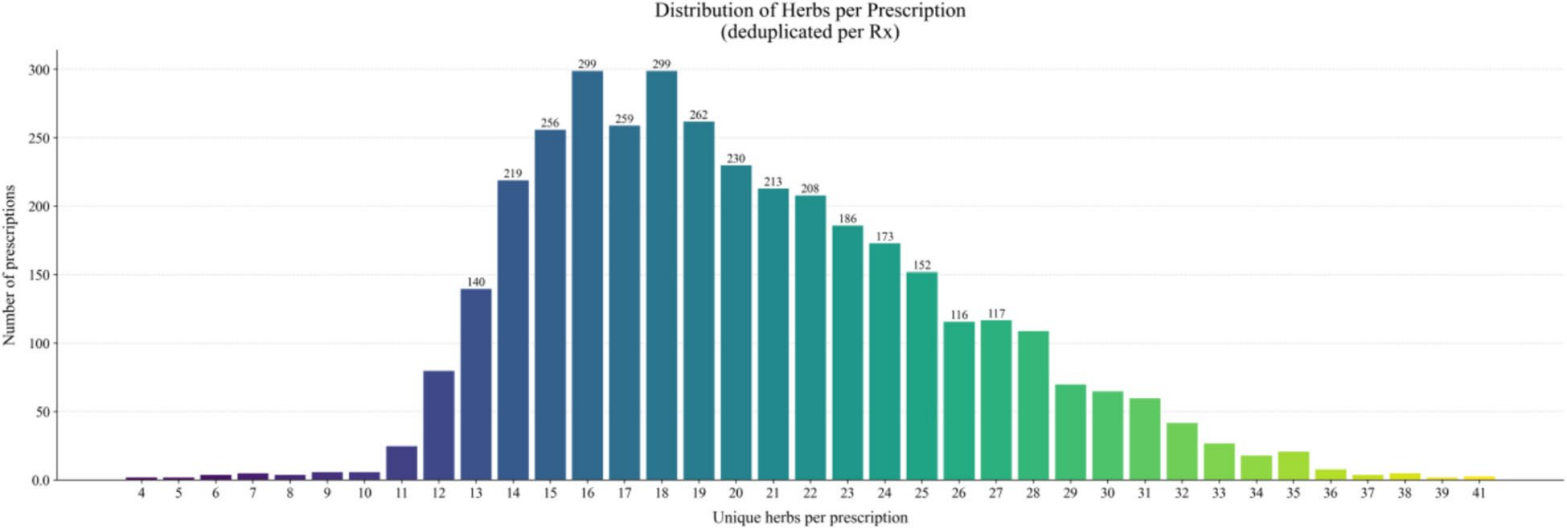
Distribution of Herbs per Prescription

The clinical case dataset was collected from authoritative institutions such as Jiangsu Provincial Hospital of Traditional Chinese Medicine and Nanjing Guoyitang Clinic. The dataset consists of real-world TCM clinical records, comprising chief complaints, symptom descriptions, TCM diagnoses, and corresponding herbal prescriptions. There were no restrictions on disease type. Moreover, approximately 3,700 high-quality medical cases were retained after filtering for first-visit records with complete information and removing duplicates and cases with missing key elements. The prescriptions in the dataset contain 520 unique herbal ingredients, with each prescription comprising an average of 20 herbs; it is particularly noteworthy that the most complex prescription contains up to 41 herbs. Figure 4 depicts the distribution of herb counts per prescription.

**Fig. 5:**
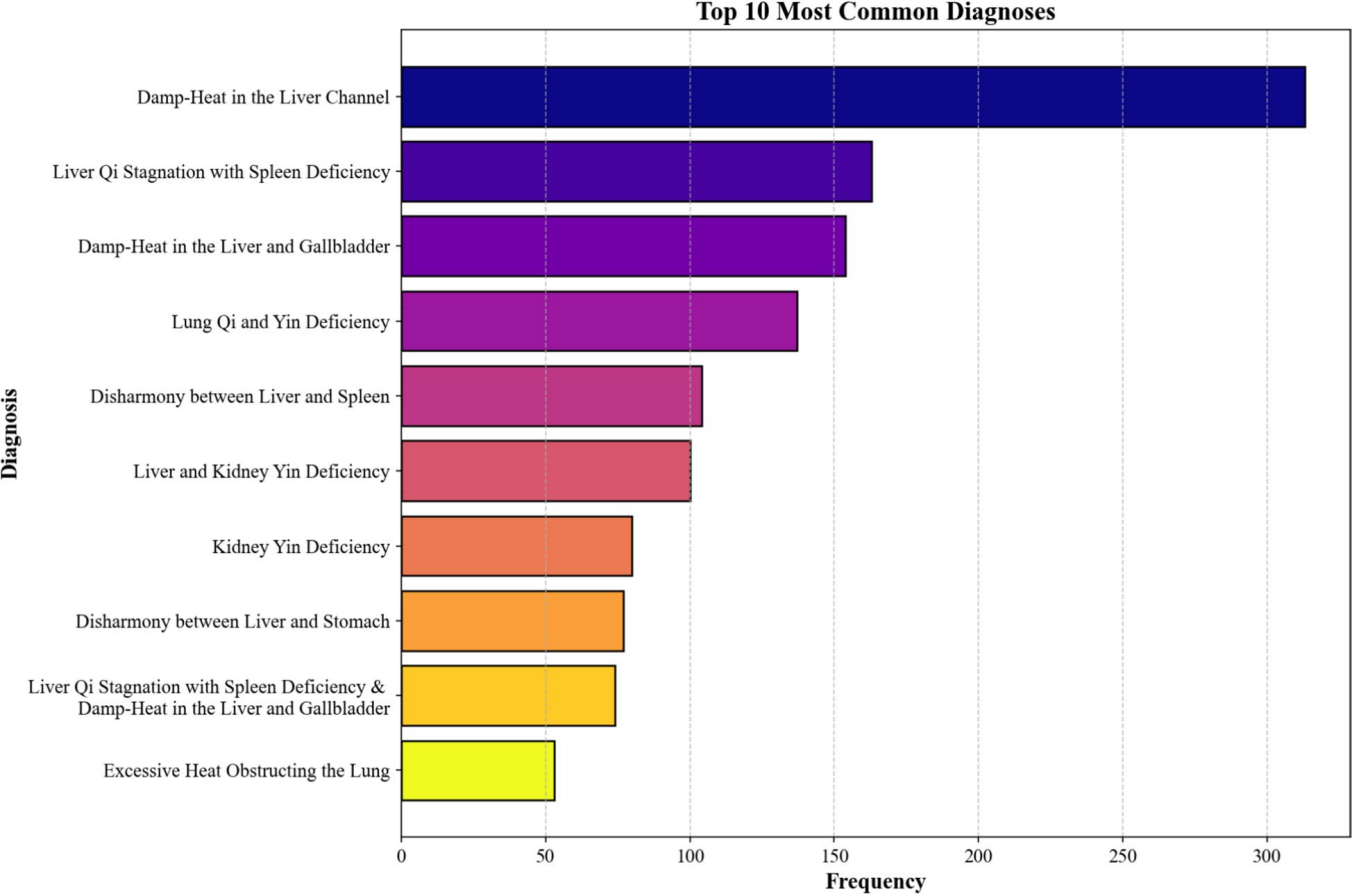
Distribution of Most Common Diagnoses

The distribution of prescription sizes exhibits a long-tail pattern: the median is 19, the interquartile range is 16–24, and the maximum is 41. Under fixed values of k,${\text{R@k < < min(1,}}\frac{{\text{k}}}{{\text{m}}}{)}$. In our full corpus, the dataset-level upper bound of recall is approximately 0.53 when k reaches 10 and around 0.91 when k is equal to 20; nonetheless, 43% of prescriptions are constrained below 1 attributable to m > 20. This phenomenon illustrates that when computing R@k, the model’s predicted prescription length may be shorter than k, which is considerably noticeable for R@30 and may give rise to comparatively lower values. Figure 5 exhibits the distribution of diagnostic frequencies in this dataset.

While the arrangement of herbs in a TCM prescription might exert insignificant effect on its therapeutic outcomes, it can considerably influence the dependability and uniformity of outputs in the context of generative modeling. For this reason, throughout the pre-processing stage, a standardized ordering strategy was applied by sorting herbs within each prescription in descending order rooted in their global frequency across the corpus. This ensured consistent structural representation of herb sequences, reinforcing the language model's ability to learn ordering patterns. The dataset was split into training and testing sets in an 8:2 ratio.

### Evaluation metric

In this section, we adopted several commonly used metrics in text generation tasks to comprehensively evaluate the accuracy and performance of the recommended model, comprising BLEU (Bilingual Evaluation Understudy) [28], ROUGE (Recall-Oriented Understudy for Gisting Evaluation) [29], P@k, R@k, and F1@k. The specific formulas are described as follows.

(1) BLEU is an extensively acknowledged metric for evaluating the quality of text generation models, which principally concentrates on precision. It measures how many n-grams in the generated text match those in the reference text. For each n-gram, the minimum count between the generated output and reference is taken, and precision ${\text{P}}_{\text{n}}$ is calculated grounded in the total number of n-grams in the candidate. The formula is given as:

$$\begin{array}{c}{Count}_{\text{clip}}=\mathit{min}\left(Count,{Max}_{{Ref}_{Count}}\right)\end{array}$$

$$\begin{array}{c}{p}_{n}=\frac{\sum_{C\in \{Candidates\}}\sum_{n-gram\in C}{\text{Count}}_{\text{clip}}\left(n-gram\right)}{\sum_{C}{}{\prime}\in \left\{{\text{Candidates}}\right\}\sum_{n-gram{\prime}\in {C}{\prime}}{\text{Count}}_{\text{clip}}\left({\text{n-gram}}{\prime}\right)}\end{array}$$

Here, Count refers to the frequency of a specific n-gram in the generated output, and ${\text{Max}}_{{\text{Ref}}_{\text{Count}}}$ defines the maximum number of times it appears in the reference text. The final BLEU score is calculated by taking the geometric mean of ${\text{P}}_{\text{n}}$ and applying a brevity penalty (BP) to prevent very short outputs from being unfairly favored. The formula is expressed as:

$$\begin{array}{c}BP=\left\{\begin{array}{lll}1,& \text{ if }& c>r\\ {e}^{\left(1-\frac{r}{c}\right)}& \text{ if }& c\le r\end{array}\right.\end{array}$$

$$\begin{array}{c}BLEU=BP*exp\left[\left(\sum_{n=1}^{N}{w}_{n}log{p}_{n}\right)\right]\end{array}$$

$${W}_{n}=1/n$$

where c denotes the length of the generated output, and r symbolizes the length of the corresponding reference.

2. ROUGE is employed to evaluate the similarity between generated and reference texts, which predominantly centers around recall. In this study, we adopt ROUGE-1, ROUGE-2, and ROUGE-L to assess model performance. The general formula for ROUGE-N is as follows:

$$\begin{array}{c}ROUGE-N=\frac{\sum_{S\in \left\{ReferencesSentences\right\}}\sum_{{gram}_{n}\in S}{Count}_{match}\left({gram}_{n}\right)}{\sum_{S\in \left\{ReferencesSentences\right\}}\sum_{{gram}_{n}\in S}Count\left({gram}_{n}\right)}\end{array}$$

3. To offer an in-depth assessment of the prescription recommendation model's performance concerning hit rate and coverage, we employed standard ranking-based evaluation metrics, encompassing P@k, R@k and F1@k. These metrics are designed to gauge the extent to which the model accurately suggests the herbs contained in the reference prescription within the top-k returned items. The formulas are given as:

$$P@k=\frac{|TOP(sc,k)\cap hc|}{k}$$

$$R@k=\frac{|TOP(sc,k)\cap hc|}{|hc|}$$

$$F1@k=2\frac{P@k*R@k}{P@k+R@k}$$

where $k$ defines the top-k recommended items, $sc$ refers to the predicted prescription, and $hc$ symbolizes the truth prescription. These assessment indicators empower us to gauge the model's proficiency in recognizing not only the "key herbs" but also the "complete herb ensemble" within a prescription. Consequently, they offer a well-rounded perspective on the model's accuracy and thoroughness. For readability, all tabular results are reported in percentage values (%).

### Baseline models

To assess the effectiveness of integrating knowledge distillation and implicit preference learning from a lightweight model into elevating prescription recommendation capabilities, we compare our recommended model against a diverse set of LLMs of varying sizes and openness on the same test set. All baseline models are used with default configurations, without additional supervised training or retrieval-augmented generation. The selected baselines are as follows.

- ① Huatuo-o1:7B [19]: It is a Chinese medical reasoning model on the basis of Qwen2.5-7B. Trained on large-scale medical literature and clinical datasets, it shows great excellence in providing medical consultations, creating clinical notes, and dealing with medical Q&A. It supports multi-turn dialogue and evidence-based reasoning, thereby rendering it suitable for clinical decision support and health management.
- ② ShenNong: Trained on an open-source TCM knowledge graph, this model adopts an entity-centric self-instruct approach to generate over 110,000 TCM-specific instruction samples, which are employed to fine-tune the model for domain-specific tasks in TCM.
- ③ Lingdan-PR [16]: On the basis of the Lingdan pre-trained language model, it is tailored for TCM recommended prescription. It can parse electronic medical record information or symptom descriptions. In addition, it can simultaneously generate herbal prescriptions that align with the principles of syndrome differentiation and treatment.
- ④ GPT-4o: It is OpenAI's flagship multi-modal general-purpose model released in 2024, natively supporting text, image, and audio inputs. In contrast to GPT-4-turbo, it delivers expedited response times and enhanced inference efficiency, all while preserving robust language comprehension, reasoning aptitude, and multi-turn dialogue proficiency.
- ⑤ DeepSeek-V3 [30]: Released in December 2024 by DeepSeek, it adopts a Mixture-of-Experts (MoE) architecture with 671 billion total parameters, 37 billion of which are active per inference. It materializes exceptional performance in mathematics, programming, and multi-lingual tasks.
- ⑥ DeepSeek-R1:70B: Rooted in LLaMA-3.3-70B-Instruct, it is further enhanced through reinforcement learning and knowledge distillation to boost its reasoning capabilities.
- ⑦ QWQ:latest:32B: It is a 32B-parameter reasoning model grounded in the Qwen series, demonstrating remarkable proficiency in multi-task handling, text generation, and comprehension.
- ⑧ LLaMA3.1-8B: Developed by Meta, it is an open-source multi-lingual model on the basis of an optimized Transformer architecture. It is trained via supervised fine-tuning (SFT) and reinforcement learning with human feedback (RLHF), which shows superior performance in reasoning, mathematical computation, and code generation.
- ⑨ TFIDF-kNN: By employing TF-IDF, case texts are represented as sparse vectors in this model, while k-nearest neighbor retrieval (K = 5) is performed with cosine similarity. Through similarity-weighted voting, the neighboring prescriptions are subsequently aggregated to generate the Top-N recommendations.
- ⑩ SimCSE-kNN: By adopting SimCSE [31], sentence embeddings are obtained to derive dense semantic representations. It is critical to note that kNN retrieval is grounded in cosine similarity. The aggregation strategy is the same as in TFIDF-kNN. SimCSE provides more suitable semantic representations, which ultimately improves retrieval performance. The model adopted here is SimCSE-bert-base.
- ⑪ FR-Post: Herb occurrence frequencies are first calculated across the training set to establish a global frequency prior. By ranking herbs according to frequency, an initial Top-N list is generated through inference. Afterwards, "herb-pair co-occurrence" rules with high lift and minimum support are automatically mined from the training prescriptions. These rules are subsequently applied for post-processing, including completion (e.g., if A appears but its strong counterpart B is missing, B is added) and re-ranking (scored by "frequency normalization + herb-pair bonus"), yielding the final prescription list.

## Experimental results

### Comparison with baseline models

**Fig. 6:**
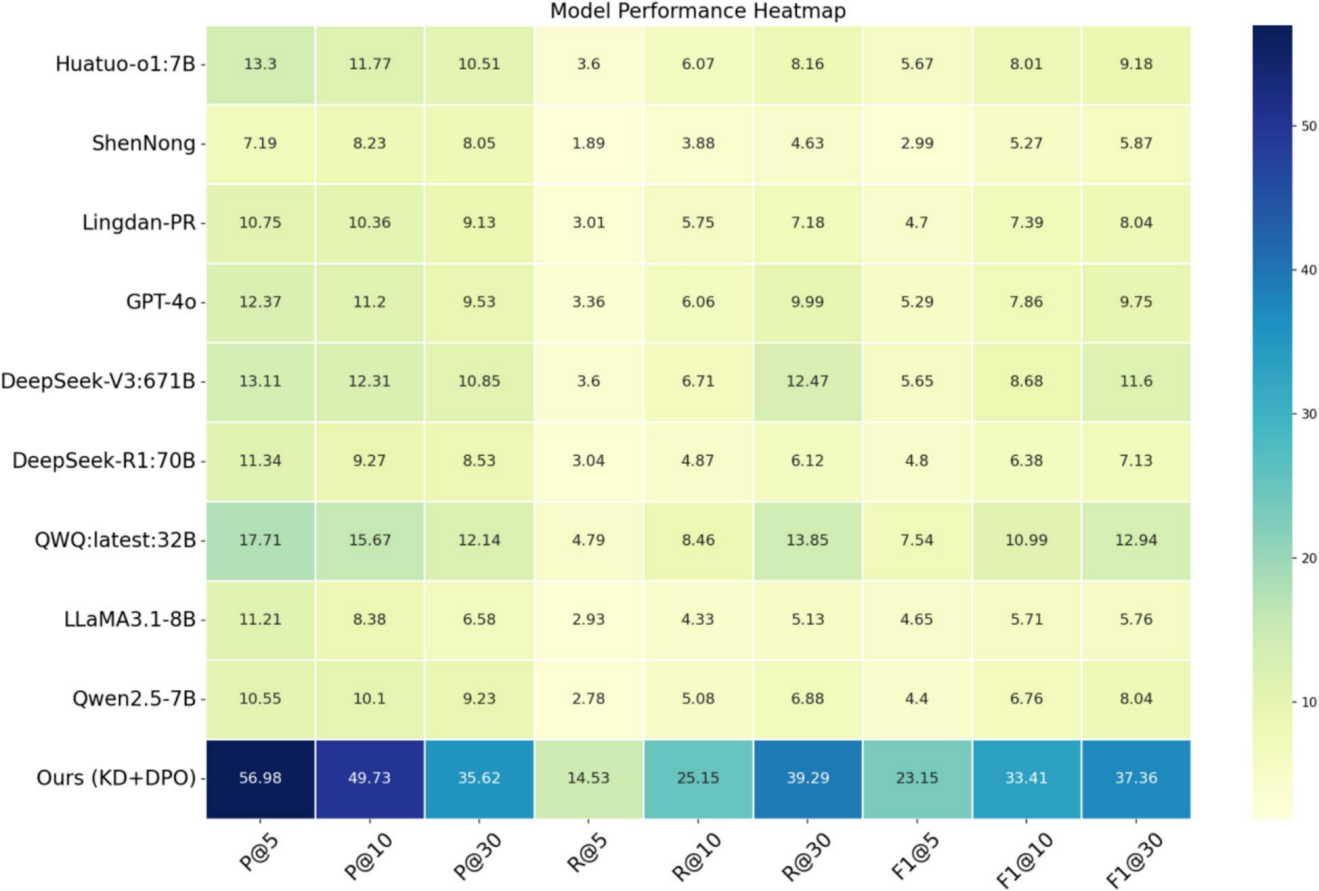
Heatmap Chart of Recommendation Performance across Models

In this section, we compare it against a set of representative open-source TCM models (e.g., Huatuo-o1:7B, ShenNong, Lingdan-PR) as well as general-purpose LLMs (e.g., GPT-4o, DeepSeek, Qwen, QWQ, LLaMA3.1) on the same test dataset, so as to systematically assess the effectiveness of the recommended method for TCM prescription recommendation. Evaluation metrics encompass Precision@k (P@k), Recall@k (R@k), and F1@k, which jointly measure the accuracy, coverage, and overall quality of prescription recommendations across a vast array of top-k settings. The detailed results are presented in Table 1 and Fig. 6.

**Table 1:** Comparison of prescription recommendation results

Model	*P@5*	*P@10*	*P@30*	*R@5*	*R@10*	*R@30*	*F1@5*	*F1@10*	*F1@30*
Huatuo-o1:7B	13.30 ± 0.29	11.77 ± 0.33	10.51 ± 0.29	3.60 ± 0.11	6.07 ± 0.21	8.16 ± 0.22	5.67 ± 0.16	8.01 ± 0.26	9.18 ± 0.25
ShenNong	7.19 ± 0.31	8.23 ± 0.41	8.05 ± 0.43	1.89 ± 0.03	3.88 ± 0.21	4.63 ± 0.24	2.99 ± 0.06	5.27 ± 0.27	5.87 ± 0.29
Lingdan-PR	10.75 ± 2.41	10.36 ± 1.42	9.13 ± 0.63	3.01 ± 0.64	5.75 ± 0.63	7.18 ± 0.64	4.70 ± 1.01	7.39 ± 0.81	8.04 ± 0.65
GPT-4o	12.37 ± 0.87	11.20 ± 0.43	9.53 ± 0.21	3.36 ± 0.21	6.06 ± 0.15	9.99 ± 0.19	5.29 ± 0.34	7.86 ± 0.23	9.75 ± 0.20
DeepSeek-V3:671B	13.11 ± 1.59	12.31 ± 1.98	10.85 ± 1.07	3.60 ± 0.46	6.71 ± 1.14	12.47 ± 1.36	5.65 ± 0.72	8.68 ± 1.44	11.60 ± 1.20
DeepSeek-R1:70B	11.34 ± 0.50	9.27 ± 0.53	8.53 ± 0.27	3.04 ± 0.15	4.87 ± 0.26	6.12 ± 0.14	4.80 ± 0.23	6.38 ± 0.35	7.13 ± 0.18
QWQ:latest:32B	17.71 ± 0.42	15.67 ± 0.14	12.14 ± 0.32	4.79 ± 0.10	8.46 ± 0.08	13.85 ± 0.28	7.54 ± 0.16	10.99 ± 0.10	12.94 ± 0.17
LLaMA3.1-8B	11.21 ± 0.30	8.38 ± 0.16	6.58 ± 0.08	2.93 ± 0.06	4.33 ± 0.04	5.13 ± 0.04	4.65 ± 0.10	5.71 ± 0.07	5.76 ± 0.05
Qwen2.5-7B	10.55 ± 0.41	10.10 ± 0.37	9.23 ± 0.72	2.78 ± 0.15	5.08 ± 0.22	6.88 ± 0.30	4.40 ± 0.23	6.76 ± 0.28	8.04 ± 0.20
Ours (KD + DPO)	**56.98 ± 0.12**	**49.73 ± 0.64**	**35.62 ± 0.42**	**14.53 ± 0.04**	**25.15 ± 0.34**	**39.29 ± 0.43**	**23.15 ± 0.06**	**33.41 ± 0.44**	**37.36 ± 0.39**

As evidently demonstrated by the above experimental findings, the suggested method achieves performance more satisfactory than that of baseline models across multiple evaluation metrics, which suggests clear improvements in overall performance. Specifically, for the P@5 metric, our model achieves 56.98%, representing an elevation of nearly 39 percentage points over the best-performing baseline model, QWQ (17.71%), which sufficiently mirrors substantial reinforcements in accuracy in recommending core herbs. It also attains 49.73% on P@10 and 35.62% on P@30, both transcending other models. With regard to recall, our model reaches 14.53%, 25.15%, and 39.29% for R@5, R@10, and R@30, respectively, generally outperforming the comparison models. This suggests that the recommended approach illustrates more favorable coverage of pivotal ingredients in actual prescriptions. The F1 scores further demonstrate enhancements in both precision and robustness. Our model achieves 23.15%, 33.41%, and 37.36% on F1@5, F1@10, and F1@30, respectively, outperforming all baselines. Notably, in F1@30, it surpasses QWQ, the best-performing baseline, by nearly 24 percentage points, which perfectly strikes a balance between accuracy and coverage.

For the baseline models, those fine-tuned on Chinese medical data, such as Huatuo-o1:7B and Lingdan-PR, suggest comparatively outstanding performance on standard metrics. In contrast, although GPT-4o and DeepSeek-V3:671B benefit from their large parameter scales, their performance is less desirable attributable to the lack of fine-tuning on TCM clinical data. The above phenomena adequately underline the significance of clinical case learning and preference alignment. Concurrently, models like ShenNong exhibit comparatively constrained performance across a wide range of metrics, evidently illustrating that existing methodologies for TCM knowledge modeling may still face challenges in handling complex and personalized prescription generation tasks.

To conclude, the recommended two-stage training strategy integrating CoT supervision by conducting knowledge distillation with implicit preference reinforcement learning guided by a lightweight model demonstrates promising potential in the TCM prescription recommendation task.

### Comparison of same-data same-setting baselines

For sake of fairness in experimental comparisons, we evaluated Qwen2.5-7B and LLaMA3.1-8B under the same knowledge distillation data and SFT training configurations. On top of that, several strong non-neural baselines underwent systematic training by utilizing prescription data to benchmark against the LLM-based models. The results are reported in Table 2.

**Table 2:** Comparison of prescription recommendation results

Model	*P@5*	*P@10*	*P@30*	*R@5*	*R@10*	*R@30*	*F1@5*	*F1@10*	*F1@30*
TFIDF-kNN	44.46 ± 0.00	39.34 ± 0.00	27.46 ± 0.00	11.17 ± 0.00	19.60 ± 0.00	40.61 ± 0.00	17.68 ± 0.00	25.81 ± 0.00	32.27 ± 0.00
SimCSE-kNN	47.63 ± 0.00	41.47 ± 0.00	28.10 ± 0.00	11.90 ± 0.00	20.58 ± 0.00	**41.41 ± 0.00**	18.84 ± 0.00	27.14 ± 0.00	32.97 ± 0.00
FR-Post	35.62 ± 0.00	31.94 ± 0.00	25.42 ± 0.00	8.73 ± 0.00	15.62 ± 0.00	37.55 ± 0.00	13.88 ± 0.00	20.69 ± 0.00	29.86 ± 0.00
LLaMA3.1-8B(KD)	55.26 ± 0.20	47.32 ± 0.32	33.19 ± 0.12	14.01 ± 0.03	23.87 ± 0.22	35.85 ± 0.75	22.36 ± 0.04	31.72 ± 0.27	34.47 ± 0.38
Qwen2.5-7B(KD)	**55.85 ± 0.36**	**48.15 ± 0.22**	**34.04 ± 0.34**	**14.18 ± 0.13**	**24.30 ± 0.13**	36.78 ± 0.57	**22.61 ± 0.19**	**32.30 ± 0.16**	**35.35 ± 0.08**

As the above analytical findings illustrate, large models fine-tuned with SFT on clinical case data consistently outperform non-neural baselines. To be specific, Qwen2.5-7B and LLaMA3.1-8B achieved F1@30 scores of 35.35% and 34.47%, respectively, both surpassing SimCSE-kNN (32.97%), TFIDF-kNN (32.27%), and FR-Post (29.86%). This suggests that LLMs fine-tuned with SFT could exhibit a more pronounced learning edge over approaches hinging exclusively on frequency priors or nearest-neighbor retrieval. It is also observed that SimCSE-kNN, which leverages dense semantic embeddings, outperforms TFIDF-kNN, which is based on sparse vector representations. Both models display comparative superiority in R@30, which is likely ascribed to the fundamental fact that fine-tuned LLMs generate complete prescriptions whose lengths may be fewer than 30 herbs, whereas kNN-based models always output exactly 30 candidates, thereby inflating their R@30 scores. In addition, the standard deviations of all kNN-based methods are reported as 0 because their retrieval and evaluation procedures are entirely deterministic — given fixed training and test sets, the value of K, TF-IDF or SimCSE embedding computations, and cosine similarity search, these models produce identical predictions across runs without any random sampling or stochastic optimization. In contrast to Qwen2.5-7B and LLaMA3.1-8B, Qwen2.5-7B consistently outperforms LLaMA3.1-8B across all metrics, though the differences are still insignificant. This edge might arise from Qwen's pretraining data aligning more tightly with Chinese clinical texts or its parameter-efficiency gains. Importantly, the comparable performance also indicates that the knowledge distillation strategy is not model-specific and exhibits favorable generalizability. For this reason, Qwen2.5-7B was selected as the backbone model for this study.

### Comparison of sovereign, minister, assistant, and courier (SMAC-based) and frequency-based prescription ordering methods

**Fig. 7:**
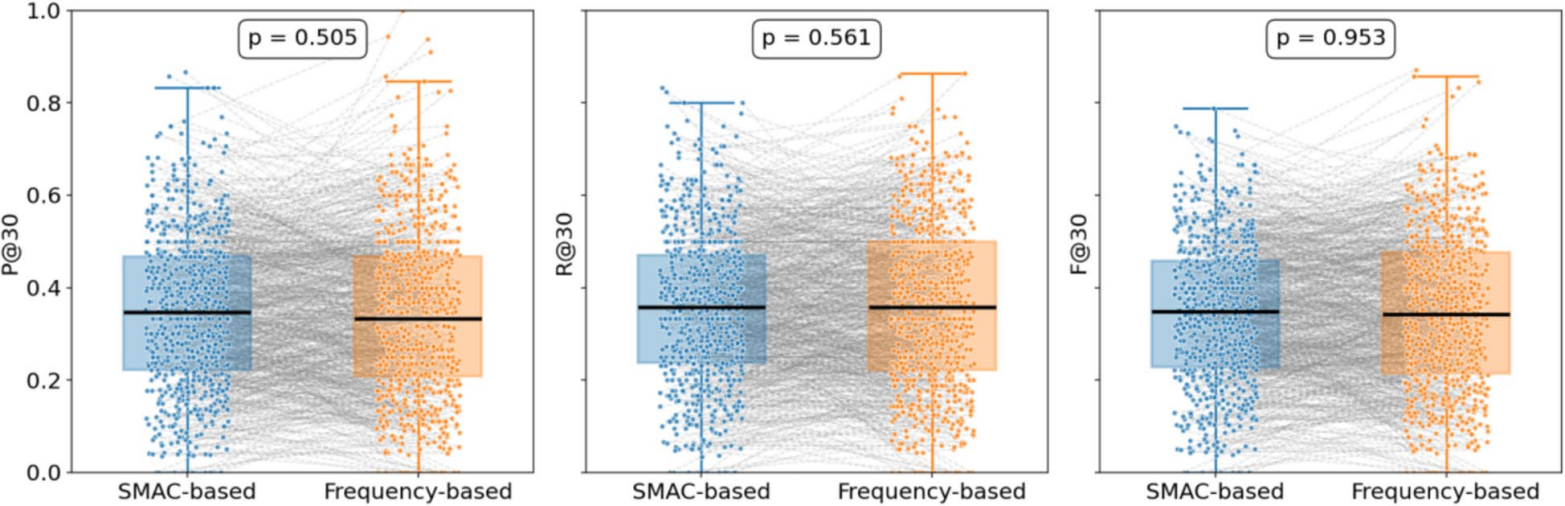
Boxplot Comparison of Prescription Recommendation Performance

In the knowledge distillation dataset, GPT-4o classified and ranked prescriptions following the sovereign-minister-assistant-courier hierarchy, and benchmarked them against a frequency-based ordering strategy (while keeping the remaining reasoning analysis identical). The outcomes are detailed in Table 3, while Fig. 7 displays pairwise comparisons and t-test results between SMAC-based and frequency-based rankings under a truncation threshold of 30.

**Table 3:** Comparison of prescription recommendation results

Fine-tuning Method	*P@5*	*P@10*	*P@30*	*R@5*	*R@10*	*R@30*	*F1@5*	*F1@10*	*F1@30*
SMAC-based	48.10 ± 0.97	42.47 ± 0.50	**34.72 ± 0.35**	12.28 ± 0.26	21.64 ± 0.26	35.76 ± 0.19	19.57 ± 0.41	28.67 ± 0.34	35.23 ± 0.27
Frequency-based	**55.85 ± 0.36**	**48.15 ± 0.22**	34.04 ± 0.34	**14.18 ± 0.13**	**24.30 ± 0.13**	**36.78 ± 0.57**	**22.61 ± 0.19**	**32.30 ± 0.16**	**35.35 ± 0.08**

The frequency-based approach outperformed under smaller truncation settings (e.g., P@5, P@10, R@5, R@10, F1@5, F1@10). This advantage may arise from frequency ordering capturing high-frequency herb patterns, making it easier for the model to correctly predict core herbs in the top-ranked positions, thereby improving both precision and recall. By contrast, the SMAC-based approach emphasizes structural and functional complementarity among herbs, which may not be fully leveraged at small truncation levels. Nonetheless, with the elevation of the truncation depth (e.g., at 30), box plots illustrate that the pairwise t-tests for P@30, R@30, and F1@30 all yield insignificant results (p = 0.505, 0.561, 0.953, respectively), indicating no statistically significant differences. It is noteworthy that the SMAC-based ordering merely hinged on GPT-4o's analysis, which may bring about instability and mis-classification. Aside from that, this ordering does not necessarily reflect physician prescribing logic. While arrangement by TCM expert could likely yield higher fidelity, it would require substantial manual effort. As a consequence, this study adopts frequency-based ordering for prescription ranking, while leaving deeper exploration of SMAC-based strategies to future work.

### Comparison of knowledge distillation results

**Fig. 8:**
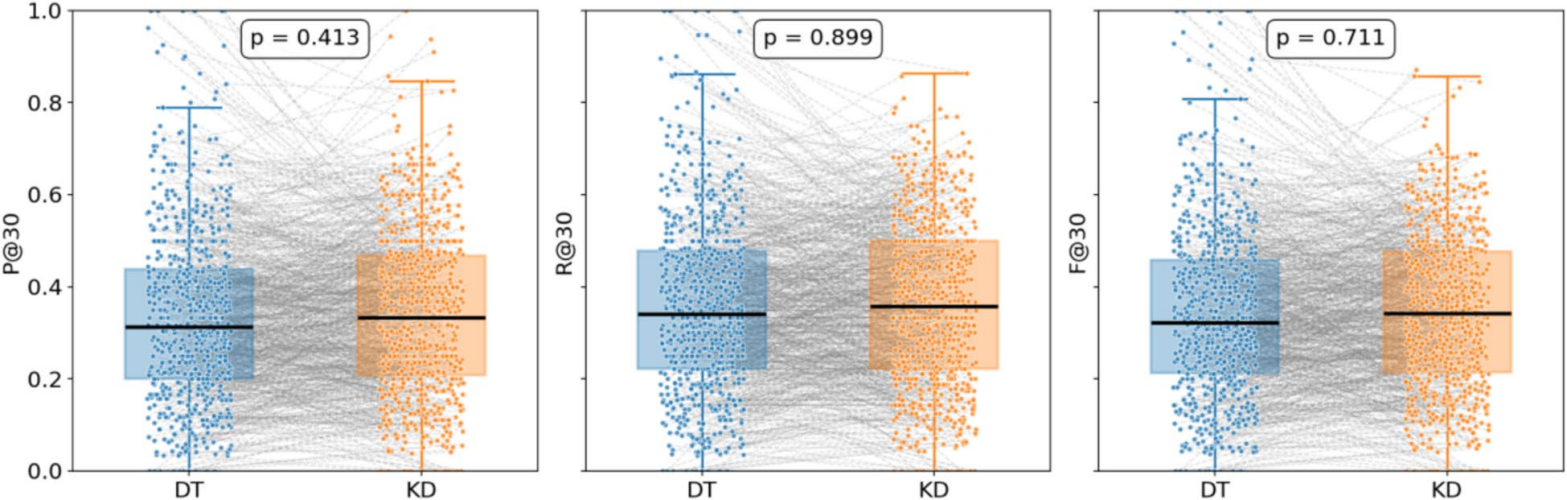
Boxplot Comparison of Prescription Recommendation Performance

With an aim to validate the efficiency of the knowledge distillation strategy in TCM prescription recommendation, we conducted a controlled experiment where three model variants were compared in a systematic manner, a model trained by utilizing CoT samples distilled from GPT-4o (Knowledge Distillation, KD), a model directly fine-tuned on the same dataset without distillation (Direct Training, DT), and the original Qwen2.5-7B model used as a performance baseline. Specific findings across all metrics are presented in Table 4 and illustrated in Fig. 8.

**Table 4:** Comparison of prescription recommendation results

Fine-tuning Method	*P@5*	*P@10*	*P@30*	*R@5*	*R@10*	*R@30*	*F1@5*	*F1@10*	*F1@30*
Qwen2.5-7B	10.55 ± 0.41	10.10 ± 0.37	9.23 ± 0.72	2.78 ± 0.15	5.08 ± 0.22	6.88 ± 0.30	4.40 ± 0.23	6.76 ± 0.28	8.04 ± 0.20
direct training	**56.71 ± 0.28**	48.07 ± 0.70	33.91 ± 0.12	**14.34 ± 0.09**	24.18 ± 0.35	36.04 ± 0.11	**22.89 ± 0.13**	32.18 ± 0.46	34.94 ± 0.11
knowledge distillation	55.85 ± 0.36	**48.15 ± 0.22**	**34.04 ± 0.34**	14.18 ± 0.13	**24.30 ± 0.13**	**36.78 ± 0.57**	22.61 ± 0.19	**32.30 ± 0.16**	**35.35 ± 0.08**

As the analytical results demonstrate, both the knowledge distillation and direct training model obtain more desirable performance than the original Qwen2.5-7B base model, which adequately illustrates that learning from clinical case data is effective in reinforcing LLM performance on TCM prescription tasks. When the truncation threshold was set to 5, the direct training model outperformed the knowledge distillation model across all metrics. Nevertheless, the knowledge distillation model consistently surpassed direct training at thresholds of 10 and 30, suggesting that direct training tends to overfit surface-level herb–symptom mappings, while distillation displays more favorable advantages in capturing underlying reasoning, thereby demonstrating more robust performance at larger truncation depths. In particular, pairwise t-tests for P@30, R@30, and F1@30 (p = 0.413, 0.899, 0.711, respectively) revealed no statistically significant differences, indicating that integrating CoT insights via knowledge distillation does not impair the model's learning or generalization in prescription recommendations.

**Fig. 9:**
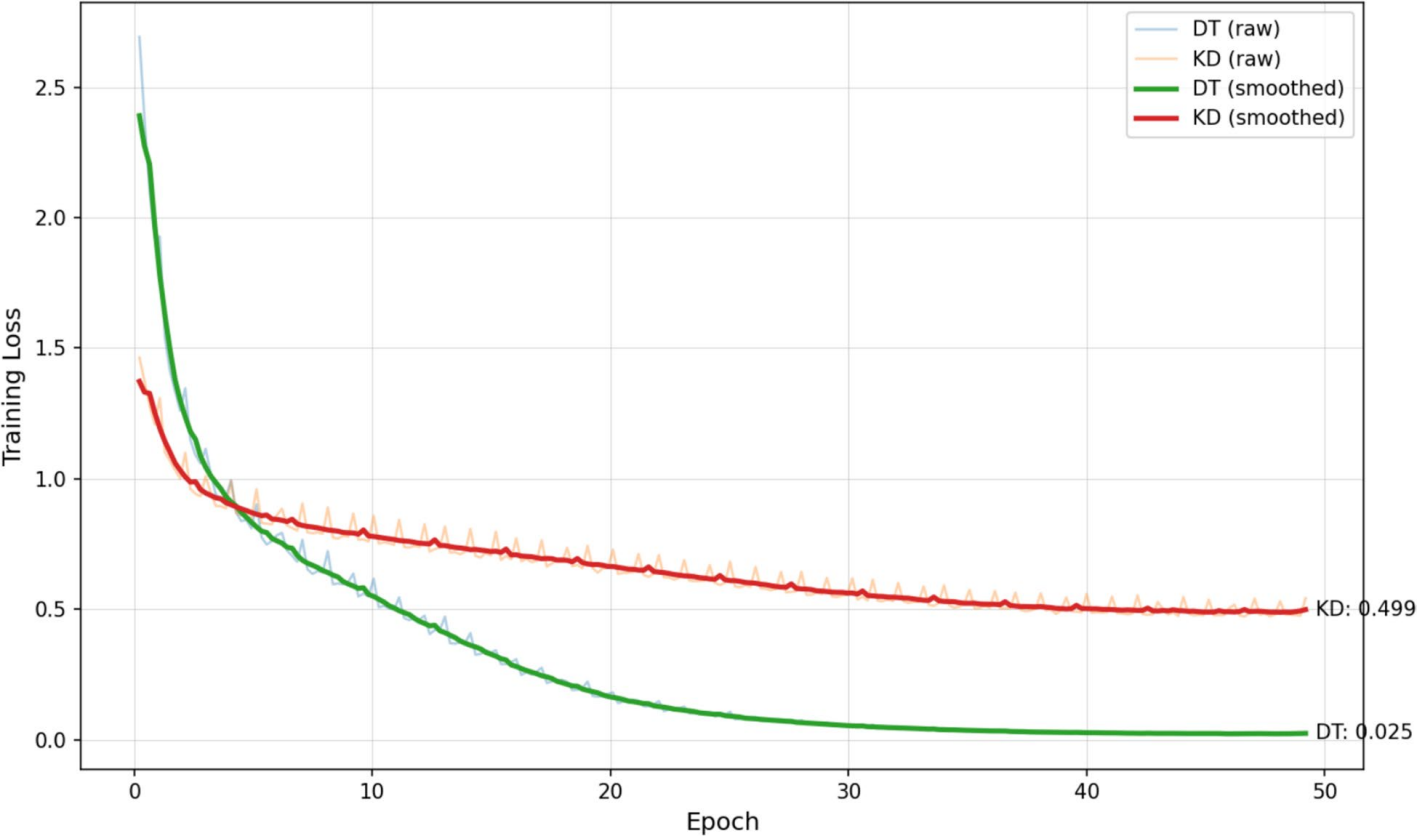
Training Loss Curves of DT and KD Methods

On top of that, we employed BLEU-4 and ROUGE scores to further evaluate the quality of generated outputs from a natural language generation perspective. The results are summarized in Table 5 and the comparison of loss curves is illustrated in Fig. 9.

**Table 5:** Comparison of supervised fine-tuning results

Fine-tuning Method	BLEU-4	ROUGE-1	ROUGE-2	ROUGE-L
direct training	30.58 ± 0.17	39.27 ± 0.08	**35.04 ± 0.11**	**51.47 ± 0.08**
knowledge distillation	**31.00 ± 0.49**	**49.72 ± 0.33**	30.47 ± 0.55	41.92 ± 0.49

As illustrated by the above findings, the knowledge distillation model performs slightly better on BLEU-4 and ROUGE-1, scoring 31.00 and 49.72 respectively, nearly 10 points higher than the direct training model on ROUGE-1. This may indicate higher lexical overlap and potentially better syntactic consistency in relation to the reference prescriptions. Additionally, it may mirror the significance of GPT-4o–generated samples, which could have guided the student model to achieve more natural and coherent language output. By contrast, the direct training model materialized higher scores on ROUGE-2 and ROUGE-L, with ROUGE-L reaching 51.47. This phenomenon suggests that the model may be better at capturing the linguistic structure and long-range alignment patterns of authentic clinical records in the training set, thereby illustrating more desirable performance on recall-oriented metrics. As evidenced in the loss curves, the direct training model eventually converged to a smoothed loss of approximately 0.025, whereas the knowledge distillation model stabilized at around 0.499. As suggested by our research findings, the direct training model is inclined to overfit the training data, triggering a lower training error; in contrast, the knowledge distillation model, guided by the teacher model, adheres to constraints that help maintain a more balanced loss. To some degree, this characteristic may contribute to its enhanced cross-institutional generalization capability.

**Fig. 10:**
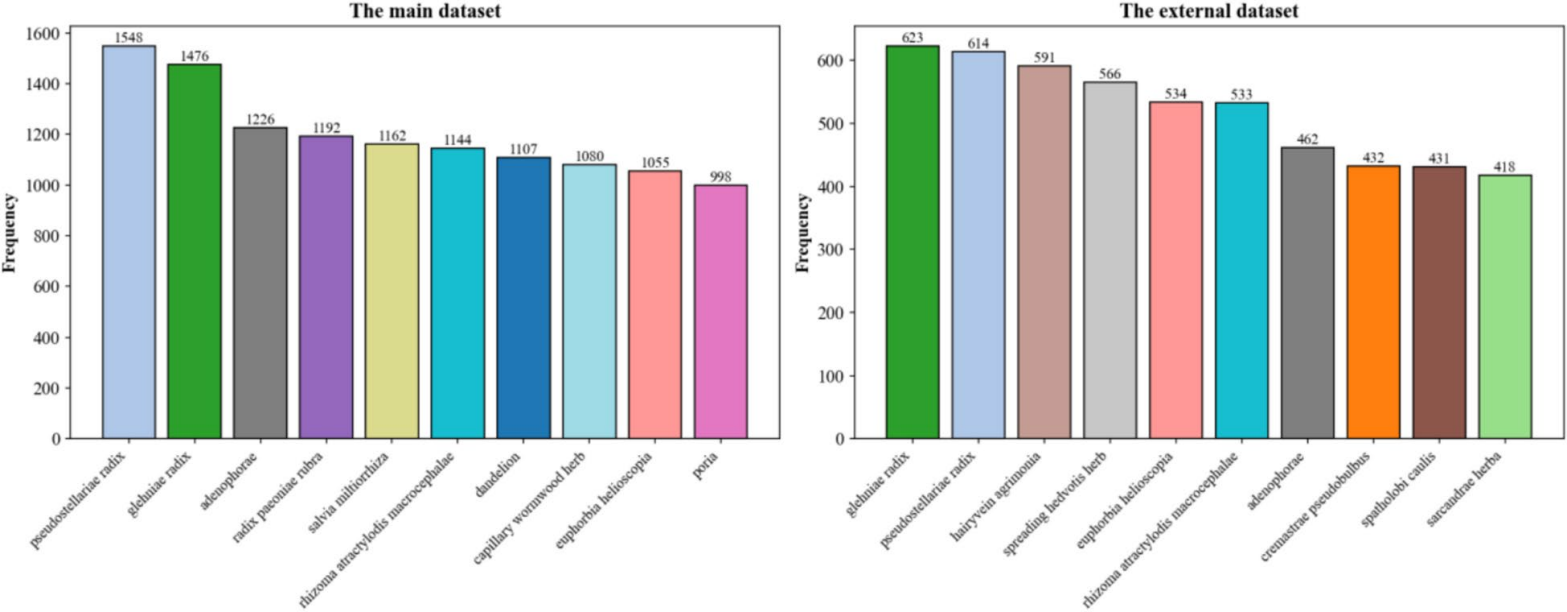
Top Frequent Herbs in The Main Dataset and The External Dataset

**Fig. 11:**
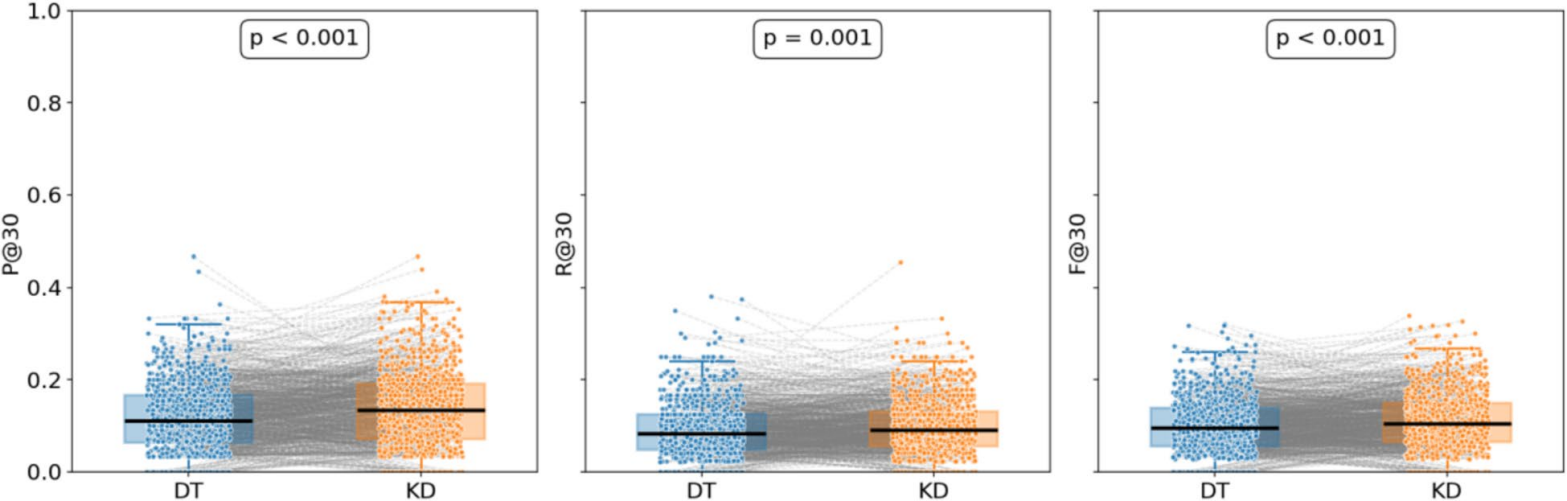
Boxplot Comparison of Prescription Recommendation Performance

With the aim of gauging the model's generalization performance in the presence of heterogeneous conditions, we further carried out an evaluation of its performance on an external cross-institution dataset. This test set comprises 919 real-world TCM clinical records not seen throughout training. The data differs substantially from the original dataset with reference to disease distribution and prescription philosophy, thus offering a robust benchmark for cross-domain transferability (Fig. 10 further illustrates striking discrepancies in herb usage between these two datasets through frequency analysis. For instance, hairyvein agrimonia and spreading hedvotis herb, both distinguished for their anti-tumor and heat-clearing detoxifying effects, are universally employed as adjuvant therapies for cancer.). Results are provided in Table 6 and Figs. 11.

**Table 6:** Comparison of prescription recommendation results

Fine-tuning Method	*P@5*	*P@10*	*P@30*	*R@5*	*R@10*	*R@30*	*F1@5*	*F1@10*	*F1@30*
direct training	21.61 ± 0.36	16.49 ± 0.11	11.86 ± 0.24	3.47 ± 0.02	5.26 ± 0.03	9.04 ± 0.11	5.98 ± 0.03	7.98 ± 0.04	10.26 ± 0.13
knowledge distillation	**21.81 ± 0.30**	**18.59 ± 0.10**	**13.49 ± 0.13**	**3.50 ± 0.09**	**5.96 ± 0.13**	**9.89 ± 0.38**	**6.03 ± 0.15**	**9.03 ± 0.16**	**11.41 ± 0.22**

As the above research findings evidenced, the knowledge distillation model exhibits performance more satisfactory than the direct training model across multiple metrics, suggesting superior generalization capability. Specifically, the knowledge distillation model outperformed the direct training model on P@30, F1@30, and R@30 by 1.63%, 0.85%, and 1.15%, separately. As demonstrated by the statistical analysis in Fig. 11, the results of paired t-tests were P@30: p < 0.001, R@30: p = 0.001, and F1@30: p < 0.001, suggesting that the knowledge distillation model achieved substantial reinforcements in generalization on external datasets. As revealed by the above findings, the CoT tripartite samples generated under GPT-4o guidance throughout the distillation process enabled the student model not only to learn surface-level herb matching but also to internalize generalizable patterns of TCM diagnostic reasoning, thereby reinforcing its capacity for generalization and cross-domain transfer.

### Results of BART implicit preference-based reinforcement learning

**Fig. 12:**
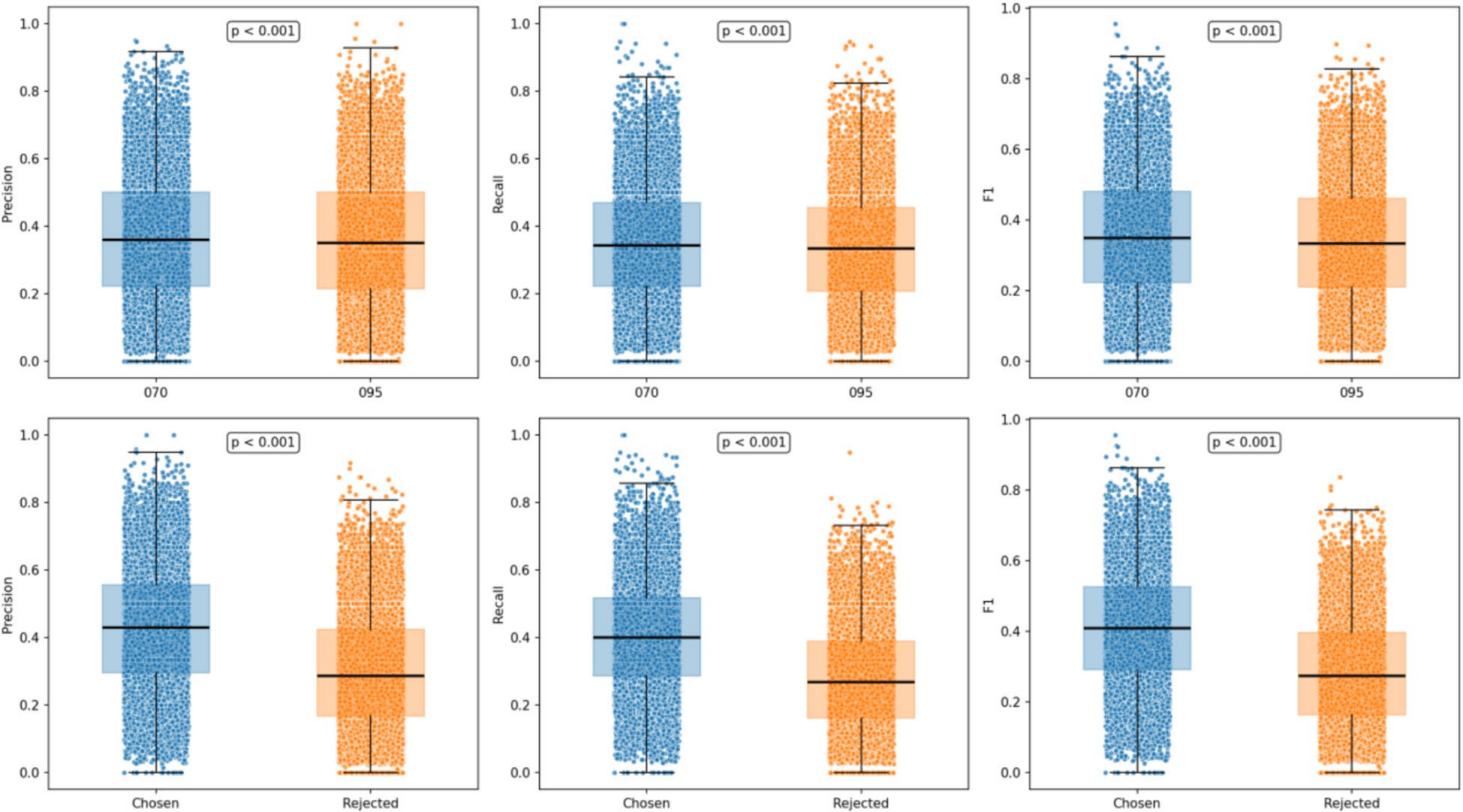
Distributional Analysis of BART-based Implicit Preference Signals

Figure 12 illustrates the data collection process for BART implicit preference.

The top three plots compare the overall prescription prediction results of the knowledge distillation model under different temperature settings (0.70 and 0.95) with the BART reference predictions, assessed by Precision, Recall, and F1. Notably, both temperature configurations exhibited statistically significant differences in the overall distributions (p < 0.001), indicating that temperature adjustments influence the diversity and certainty of the generated prescriptions. The bottom three plots show the sample-level comparisons between the Chosen and Rejected groups against BART predictions. The Chosen group consistently surpassed the Rejected group across all three metrics (p < 0.001), suggesting the model may better distinguish high- from low-quality outputs, though further validation is essential.

**Fig. 13:**
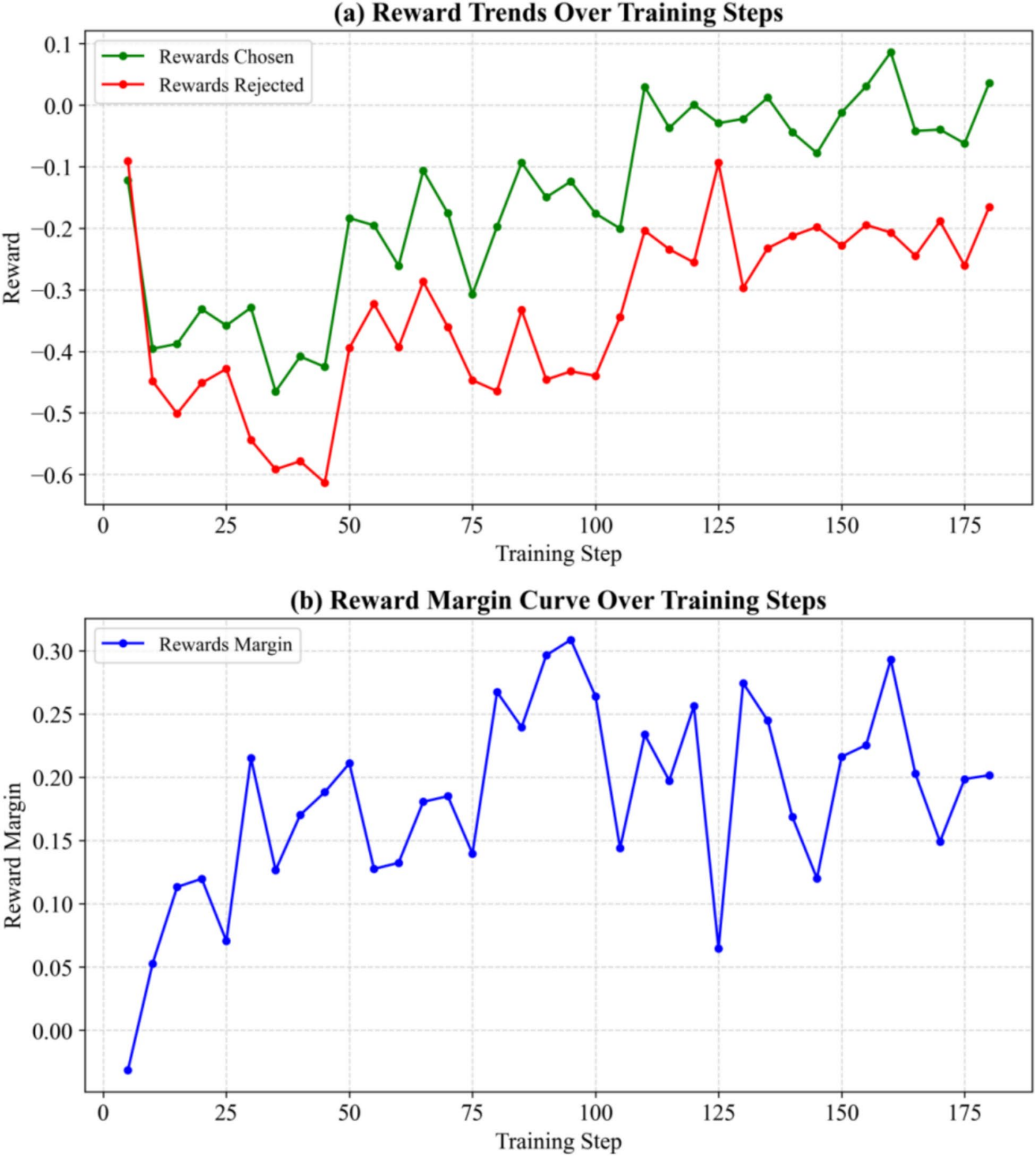
Reward Parameter Trends During DPO Reinforcement Learning

Following the collection of BART implicit preference data, the knowledge distillation model was further trained by adopting the DPO method. The reward dynamics during training are presented in Fig. 13.

As revealed by the above findings, DPO effectively maintained higher reward values for Rewards Chosen samples and lower values for Rewards Rejected samples, illustrating that DPO can help the model differentiate between superior and inferior outputs. In addition, Rewards Margin remained at relatively high levels, further validating the effectiveness of this method in aligning model predictions with implicit preferences.

**Fig. 14:**
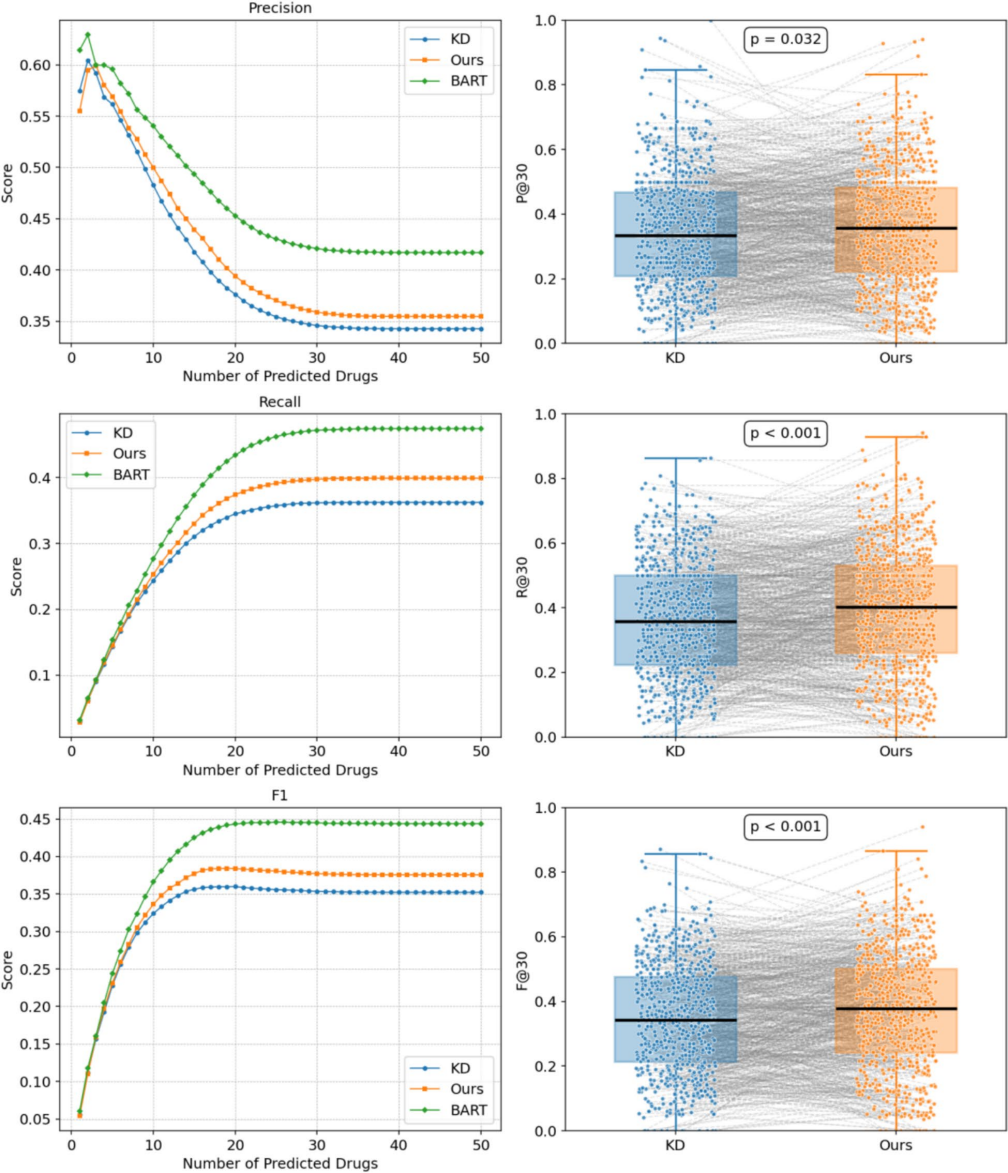
Boxplot Comparison of Prescription Recommendation Performance

**Fig. 15:**
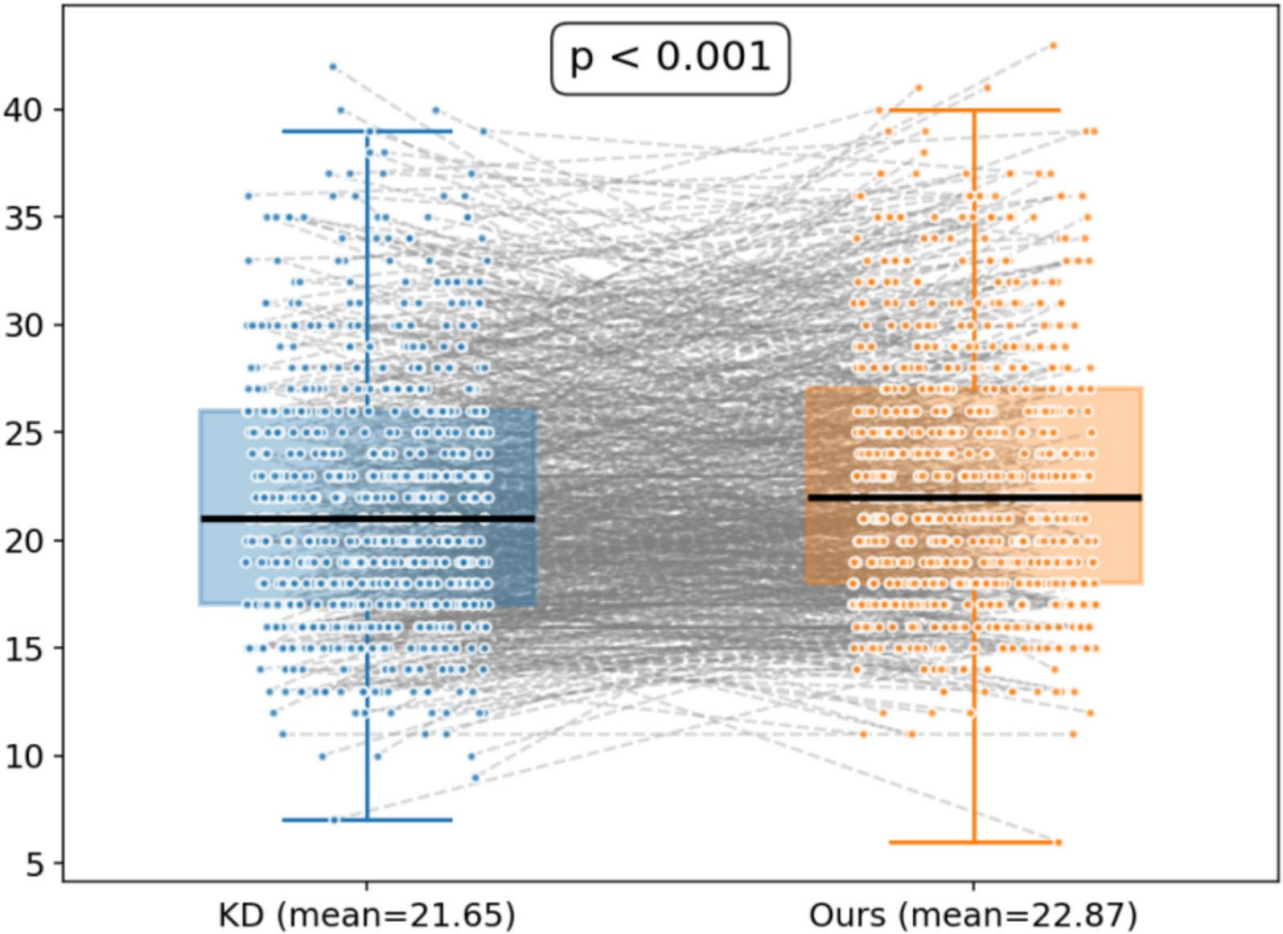
Boxplot Comparison of Prescription Length

In order to assess the effect of reinforcement learning guided by implicit preferences on TCM prescription recommendation, we proceeded to carry out a comparative analysis. During this process, we compared our ultimate recommended model (KD + DPO) with two other models. For one thing, there is the knowledge distillation model. For another, we have the BART model, which was trained solely based on the symptom-to-prescription mapping. The results are presented in Table 7 and Figs. 14, 15.

**Table 7:** Comparison of prescription recommendation results

Fine-tuning Method	*P@5*	*P@10*	*P@30*	*R@5*	*R@10*	*R@30*	*F1@5*	*F1@10*	*F1@30*
BART	**59.32 ± 0.64**	**54.05 ± 0.42**	**42.17 ± 0.12**	**15.27 ± 0.15**	**27.64 ± 0.20**	**47.36 ± 0.16**	**24.28 ± 0.24**	**36.57 ± 0.27**	**44.61 ± 0.11**
knowledge distillation	55.85 ± 0.36	48.15 ± 0.22	34.04 ± 0.34	14.18 ± 0.13	24.30 ± 0.13	36.78 ± 0.57	22.61 ± 0.19	32.30 ± 0.16	35.35 ± 0.08
Ours(KD + DPO)	56.98 ± 0.12	49.73 ± 0.64	35.62 ± 0.42	14.53 ± 0.04	25.15 ± 0.34	39.29 ± 0.43	23.15 ± 0.06	33.41 ± 0.44	37.36 ± 0.39

Altogether, the BART model attained top scores across most evaluation metrics, demonstrating notably robust performance to generate accurate TCM prescriptions in comparison with larger models. This superior performance can be attributed to the following two key factors. First and foremost, BART adopts a classic encoder–decoder architecture and performs well on Seq2Seq tasks, thereby rendering it particularly effective at capturing semantic relationships between input symptoms and output prescriptions. Apart from that, with a relatively small parameter size of 406 M, the BART model demonstrates superior training and inference efficiency in comparison to LLMs. This characteristic not only enables it to undergo comprehensive fine-tuning even when computational resources are constrained, but also may empower the model to acquire symptom–prescription mappings from medical case data in a more efficient manner. Nonetheless, despite its favourable performance, BART lacks diagnostic reasoning and explainability, rendering it unsuitable for direct deployment in trustworthy prescription recommendation systems. Instead, it serves as a better preference generator in the reinforcement learning phase, offering guidance to LLMs by providing high-quality reference outputs. This facilitates a more precise alignment in the process of reinforcement tuning, thereby contributing to an elevation in the final recommendation accuracy of the large model.

In comparison, the recommended KD + DPO model outperformed the standalone knowledge distillation model on nearly all metrics, which facilitates the most remarkable increase of approximately 1.58% in P@30(35.62%), 2.51% in R@30 (39.29%) and 2.01% in F1@30 (37.36%). As suggested in the statistical analysis in Fig. 14, the paired t-test results were P@30: p = 0.032, R@30: p < 0.001, and F1@30: p < 0.001, revealing statistically significant improvements. On average, the KD + DPO model produced prescriptions roughly one herb longer than the KD model, with this difference achieving statistical significance. These enhancements illustrate that the implicit preferences provided by BART contributed positively to optimization throughout the reinforcement learning stage. While the KD + DPO model still falls slightly short of BART in absolute performance, it demonstrates a more favorable balance between prescription accuracy and explainability, which exhibits enormous potential for future clinical applications.

**Fig. 16:**
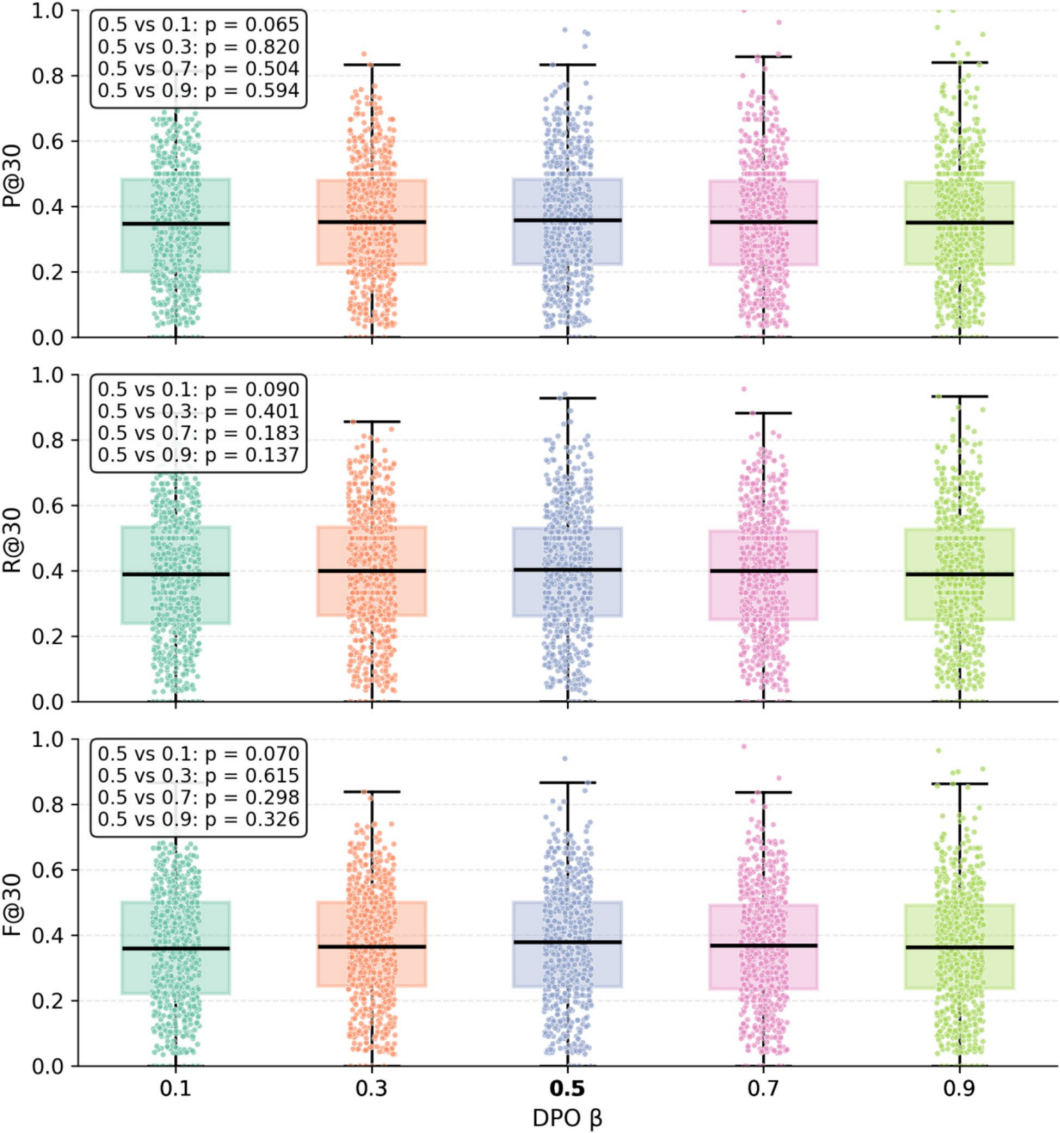
Boxplot Comparison of Prescription Recommendation Performance

In DPO, the scaling coefficient β conducts a critical role in preference alignment. To assess the impact of β on training outcomes, we conducted comparative experiments with β values set to 0.1, 0.3, 0.5, 0.7, and 0.9, separately. The corresponding results are presented in Table 8 and Fig. 16.

**Table 8:** Comparison of prescription recommendation results

β	*P@5*	*P@10*	*P@30*	*R@5*	*R@10*	*R@30*	*F1@5*	*F1@10*	*F1@30*
0.1	55.14 ± 0.06	48.50 ± 0.08	34.90 ± 0.19	14.43 ± 0.02	24.73 ± 0.05	39.09 ± 0.34	22.96 ± 0.03	32.76 ± 0.06	36.88 ± 0.26
0.3	56.37 ± 0.29	49.51 ± 0.18	35.37 ± 0.31	14.39 ± 0.09	25.07 ± 0.07	39.13 ± 0.38	22.92 ± 0.14	33.29 ± 0.10	37.16 ± 0.33
0.5	56.98 ± 0.12	**49.73 ± 0.64**	**35.62 ± 0.42**	14.53 ± 0.04	**25.15 ± 0.34**	**39.29 ± 0.43**	23.15 ± 0.06	**33.41 ± 0.44**	**37.36 ± 0.39**
0.7	**57.49 ± 0.25**	49.52 ± 0.35	35.19 ± 0.17	**14.61 ± 0.03**	25.00 ± 0.17	38.71 ± 0.20	**23.30 ± 0.06**	33.23 ± 0.22	36.86 ± 0.18
0.9	56.45 ± 0.44	49.05 ± 0.27	35.01 ± 0.39	14.40 ± 0.12	24.81 ± 0.15	38.43 ± 0.33	22.95 ± 0.19	32.95 ± 0.19	36.64 ± 0.36

β serves as a scaling factor that regulates the intensity of preference signals during DPO training. An excessively small value could underweight preference signals, making the model resemble supervised learning and impeding proper preference alignment. Conversely, an excessively large β may lead the model to overfit to preference signals during training, thereby undermining its generalization capability. As the analytical outcomes suggest, the model achieves relatively superior performance across precision, recall, and F1 metrics when β is equal to 0.5, demonstrating that a moderate value of β strikes a more satisfactory balance between preference alignment and generalization. The statistical analysis in Fig. 16 further reveals that all paired t-test results exceeded 0.05, suggesting the model's robustness remains intact within an appropriate parameter range. Nonetheless, the overall trend suggests that β = 0.5 yielded comparatively better performance across multiple metrics. For this reason, this value was adopted as the default configuration.

**Fig. 17:**
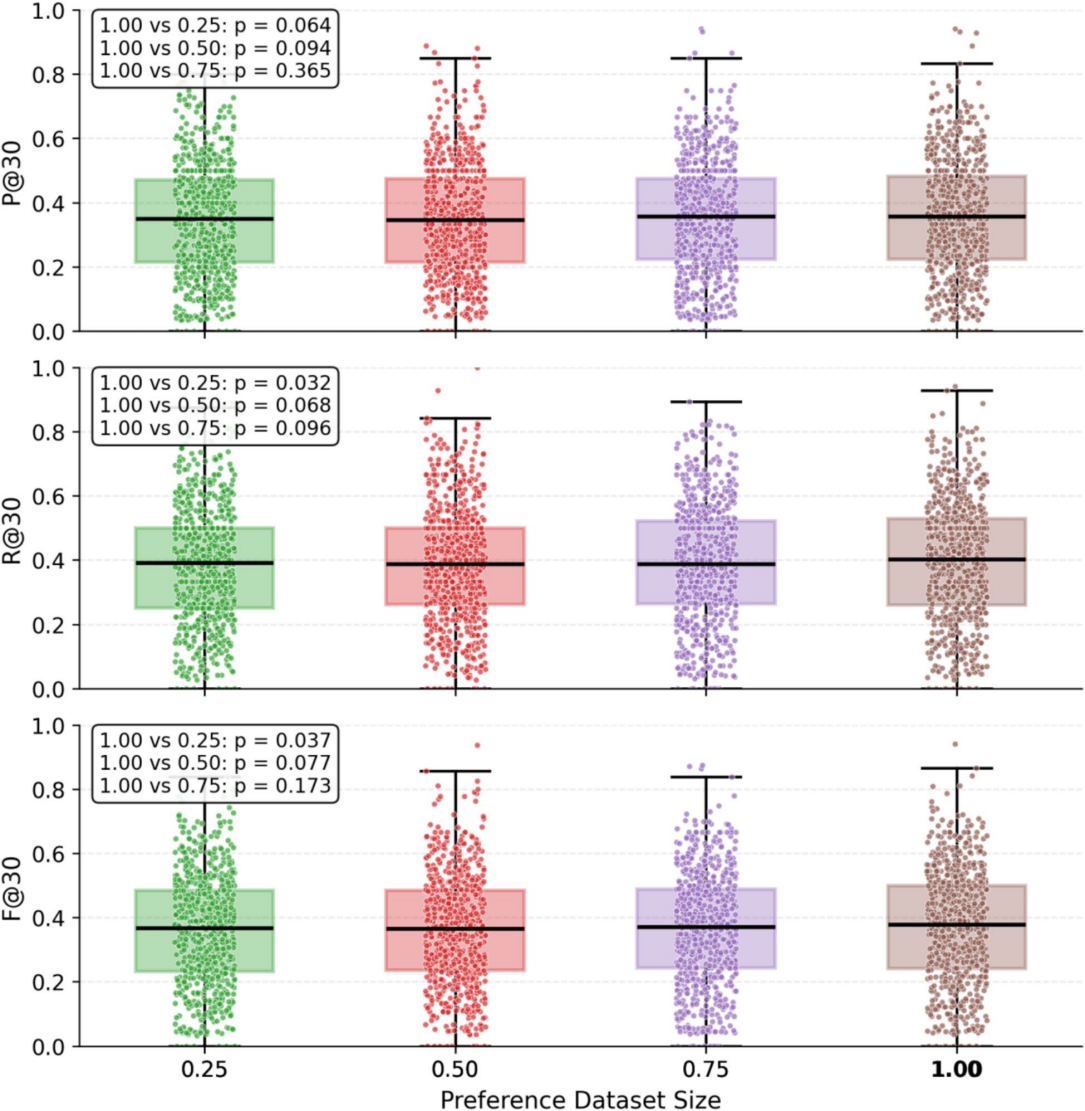
Boxplot Comparison of Prescription Recommendation Performance

To delve into how preference data scale affects DPO training outcomes, we partitioned the full preference dataset into 25%, 50%, 75%, and 100% subsets and conducted experiments with β fixed at 0.5. The results are displayed in Table 9 and Fig. 17.

**Table 9:** Comparison of prescription recommendation results

Dataset Size	*P@5*	*P@10*	*P@30*	*R@5*	*R@10*	*R@30*	*F1@5*	*F1@10*	*F1@30*
25%	56.54 ± 0.59	48.97 ± 0.36	34.89 ± 0.40	14.37 ± 0.18	24.72 ± 0.15	38.44 ± 0.21	22.92 ± 0.27	32.85 ± 0.21	36.58 ± 0.30
50%	56.37 ± 0.69	48.79 ± 0.49	34.50 ± 0.42	14.37 ± 0.21	24.69 ± 0.30	38.35 ± 0.26	22.90 ± 0.32	32.79 ± 0.37	36.32 ± 0.30
75%	56.31 ± 0.23	49.19 ± 0.29	35.19 ± 0.12	14.34 ± 0.04	24.88 ± 0.15	38.58 ± 0.22	22.86 ± 0.07	33.05 ± 0.20	36.81 ± 0.16
100%	**56.98 ± 0.12**	**49.73 ± 0.64**	**35.62 ± 0.42**	**14.53 ± 0.04**	**25.15 ± 0.34**	**39.29 ± 0.43**	**23.15 ± 0.06**	**33.41 ± 0.44**	**37.36 ± 0.39**

The findings reveal that as the data scale exceeds 0.50, precision, recall, and F1 scores steadily rise. When 100% of the preference dataset was used (i.e., fourfold data augmentation on training symptoms), the model achieved the best overall performance across all metrics, which suggests that larger-scale preference datasets may bolster the model's alignment with preference signals and elevate its prescription recommendation performance. Further paired t-test results demonstrated that the differences between the 100% and 25% data settings were statistically significant for R@30, and F1@30 (p < 0.05), while differences with 50% and 75% datasets were non-significant, despite an overall upward performance trend. These results suggest that moderately larger-scale preference training datasets enhance model performance.

### Case study

By employing a representative test sample, we conducted a case study to further evaluate the prescription recommendation capability of the suggested model in real-world TCM clinical scenarios. The selected case describes a patient with "The patient's liver function tests remain unstable, with ALT at 149, AST at 174, and GGT at 185. Subjectively, the patient reports favorable overall condition, with no hepatic pain but a sensation of discomfort beneath the costal region. Bowel movements occur daily. Additional findings include dry mouth with a slightly bitter taste. Tongue inspection shows a dark body with a yellow greasy coating, and the pulse is wiry, fine, and slippery". We compared the prescription recommendations generated by a wide spectrum of models under the same input conditions. The models include: the real prescription, the BART model (direct training), the Qwen2.5-7B direct training model, the knowledge distillation (KD) model, and the final model suggested in this study (KD + DPO). Table 10 demonstrates an all-round analysis of their outputs against the reference prescription.

**Table 10:** Comparison of case analysis

Model	Output
Real prescription	Dandelion, capillary wormwood herb, radix bupleuri, curcumae radix, sedum sarmentosum, parched scutellariae radix, magnolia bark, parched rhizoma atractylodis, amur corktree bark, phyllanthus urinaria, herba oxalidis corniculatae, sophora flavescens, rhubarb preparata, Christina loosestrife, fructus gardeniae, gentianae radix et rhizoma
Lingdan-base	In line with your description, your condition may be attributable to liver qi stagnation and damp-heat retention. The following treatment is recommended. Herbal Medicine Treatment: 1. Modified Xiaoyao Powder: bupleurum, white peony root, fructus aurantii, macrocephalae rhizoma, poria, liquorice, etc. One dose daily, decocted in water. 2. Modified Capillary Wormwood Herb Decoction:capillary wormwood herb, cape jasmine fruit, rhei radix et rhizoma, liquorice, etc. 3. Modified Gentian Liver-Draining Decoction: gentianae radix et rhizoma, scutellariae radix, cape jasmine fruit, dried rehmannia root, Chinese angelica, white peony root, etc. Acupuncture Therapy: Points such as Ganyu, Piyu, Zusanli, and Taichong may be selected, with each session involving 20 min of needle retention, once daily. Dietary Adjustment: A light diet is advised, avoiding spicy and irritating foods. Drink more water, eat fresh vegetables and fruits, and maintain adequate sleep and a positive mindset. Appropriate Rest: Avoid overexertion, engage in moderate physical exercise, and enhance overall immunity. If you experience any discomfort or doubts, please consult your physician promptly
BART	radix paeoniae rubra, dandelion, capillary wormwood herb, radix bupleuri, curcumae radix, sedum sarmentosum, parched rhizoma atractylodis, amur corktree bark, phyllanthus urinaria, prunellae spica, herba oxalidis corniculatae, sophora flavescens, abrux herb, rhubarb preparata, herba agastaches, Christina loosestrife, fructus gardeniae, all-grass of madras grangea, gentianae radix et rhizoma, reed rhizome, spora lygodii, verbenae herba
Direct training	pseudostellariae radix, radix paeoniae rubra, salvia miltiorrhiza, parched macrocephalae rhizoma, dandelion, capillary wormwood herb, radix bupleuri, poria, processed rhizoma cyperi, sedum sarmentosum, parched scutellariae radix, magnolia bark, phyllanthus urinaria, prunellae spica, parched liquorice, imperatae rhizoma, lilium davidii, parched crataegi fructus, parched medicated leaven
Knowledge distillation	*Patient Symptom Analysis* The patient presents with fluctuating liver function indices, comprising elevated ALT, AST, and GGT, indicating possible hepatic injury or inflammation. From a clinical perspective, the patient reports no liver pain but experiences hypochondriac discomfort and a slightly dry, bitter taste in the mouth. Tongue examination shows a dark tongue body with a yellow greasy coating, and the pulse is wiry, slippery, and fine. These manifestations suggest damp-heat accumulation in the liver channel. Damp-heat obstructs the liver, leading to impaired hepatic function, which manifests as hypochondriac discomfort and bitter taste, while also stagnating qi movement and disrupting normal bowel evacuation *Recommended Prescription* radix paeoniae rubra, dandelion, capillary wormwood herb, radix bupleuri, curcumae radix, sedum sarmentosum, parched scutellariae radix, magnolia bark, herba oxalidis corniculatae, abrux herb, herba agastaches, Christina loosestrife, roasted tsaoko fructus, parched corydalis rhizoma, rhizoma atractylodis *Prescription Explanation* 1. Clearing Heat and Eliminating Dampness: capillary wormwood herb, dandelion, sedum sarmentosum, Christina loosestrife, abrux herb — employed to dispel damp-heat from the liver channel. 2. Soothing the Liver and Regulating Qi: radix bupleuri, curcumae radix, parched corydalis rhizoma — to relieve liver qi stagnation, regulate qi, alleviate pain, and ease hypochondriac discomfort. 3. Clearing Heat and Detoxification: dandelion, sedum sarmentosum—to eliminate pathogenic heat and toxins, thereby improving liver function abnormalities. 4. Strengthening the Spleen and Resolving Dampness: rhizoma atractylodis, magnolia bark, herba agastaches — to fortify the spleen, resolve damp turbidity, and restore digestive balance. 5. Invigorating Blood and Dispelling Stasis: radix paeoniae rubra, parched corydalis rhizoma — to promote blood circulation and ameliorate the dark tongue coloration. 6. Harmonizing the liver and spleen: roasted tsaoko fructus, parched scutellariae radix — to harmonize liver and spleen functions, enhancing the overall therapeutic effect. This prescription primarily focuses on clearing heat and eliminating dampness, complemented by soothing the liver, detoxifying heat, strengthening the spleen, invigorating blood circulation, and harmonizing the liver and spleen, thus targeting the clinical manifestations of damp-heat accumulation in the liver channel
Ours: KD + DPO	*Patient Symptom Analysis* The patient presents with fluctuating liver function indices, accompanied by mild subjective symptoms but noticeable discomfort in the hepatic region, along with dry mouth and a slightly bitter taste. Bowel movements are normal. Tongue inspection reveals a dark tongue body with a yellow greasy coating, and the pulse is wiry, fine, and slippery. These clinical signs indicate the presence of damp-heat obstructing the liver and gallbladder. Such a pattern is commonly manifested by hepatic discomfort, bitter taste in the mouth, and a yellow greasy tongue coating. In this case, the patient's liver function abnormalities are closely associated with the damp-heat pattern of the liver and gallbladder *Recommended Prescription* radix paeoniae rubra, dandelion, capillary wormwood herb, radix bupleuri, curcumae radix, processed rhizoma cyperi, sedum sarmentosum, phyllanthus urinaria, alismatis rhizoma, prunellae spica, herba oxalidis corniculatae, sophora flavescens, rhubarb preparata, fructus gardeniae, crataegi fructus, gentianae radix et rhizoma, dried liquorice *Prescription Explanation* 1. Clearing Heat and Eliminating Dampness: capillary wormwood herb, dandelion, sedum sarmentosum, alismatis rhizoma, phyllanthus urinaria, herba oxalidis corniculatae — to dispel damp-heat and alleviate hepatic and gallbladder dysfunction. 2. Soothing the Liver and Regulating Qi: radix bupleuri, curcumae radix, processed rhizoma cyperi — to soothe the Liver, regulate qi, and relieve hepatic discomfort. 3. Invigorating Blood and Dispelling Stasis: radix paeoniae rubra, crataegi fructus — to promote blood circulation and mitigate functional abnormalities of the liver. 4. Clearing Heat and Detoxification: sophora flavescens, prunellae spica, gentianae radix et rhizoma, fructus gardeniae — to clear heat, detoxify, and further reduce damp-heat in the liver and gallbladder. 5. Unblocking the Bowels and Draining Heat: rhubarb prepared — to purge internal heat and assist in eliminating retained damp-heat. 6. Harmonizing the Prescription: dried liquorice — to moderate the properties of the other herbs and reduce potential harshness. This prescription emphasizes clearing heat and eliminating dampness as the primary therapeutic principle, supplemented by soothing the liver, invigorating blood circulation, detoxifying heat, and harmonizing the prescription, thereby addressing the manifestations of the damp-heat pattern in the liver and gallbladder

As demonstrated by the above findings, the BART model and the direct training model only output the prescription sequence, without any structured symptom analysis or interpretive explanation. This limits their explainability and transparency in clinical TCM decision-making. Among them, the BART model realized higher prescription hit rates, indicating its ability to effectively capture symptom-to-herb mappings throughout training. In contrast, both the knowledge distillation model and our suggested KD + DPO model generated complete outputs that encompass structured symptom analysis, the recommended prescription, and a reasoning chain for prescription interpretation. These models exhibit more conspicuous explainability and more satisfactory alignment with traditional TCM diagnostic reasoning processes, making them potentially more reliable in practical clinical contexts. As suggested by the above research findings, the model's prescription recommendations exhibit noticeably improved accuracy after reinforcement learning, suggesting that the implicit preferences provided by the BART model could potentially have fulfilled a constructive function in steering the optimization process, thereby ameliorating recommendation performance without sacrificing explainability. The Lingdan-based LLM demonstrated comparatively superior accuracy in syndrome differentiation, diagnostic analysis, and explanation of formula usage, and additionally generated recommendations for acupuncture treatment. Nonetheless, in recommended prescription, it was primarily dependent on classical prescription with minor modifications tailored to the patient's condition. Despite the fundamental fact that this approach presented a degree of rationality, it still exhibited certain limitations compared with our proposed model, particularly in terms of herb-symptom matching accuracy and the integration of reasoning chains with patient's symptoms.

To sum up, the BART model presents noticeable drug matching accuracy, thereby rendering it favorably suited as a source of implicit preferences for data construction in the reinforcement learning stage; the knowledge distillation model generates comparatively well-structured reasoning chains, which apparently mirrors consistent linguistic logic and elements of TCM diagnostic thinking, in spite of its relatively lower recommendation accuracy; by contrast, the recommended KD + DPO model integrates the strengths of both approaches, ultimately attaining a comparatively balanced performance across recommendation accuracy, reasoning architecture, and interpretability.

### Expert evaluation and error analysis

**Fig. 18:**
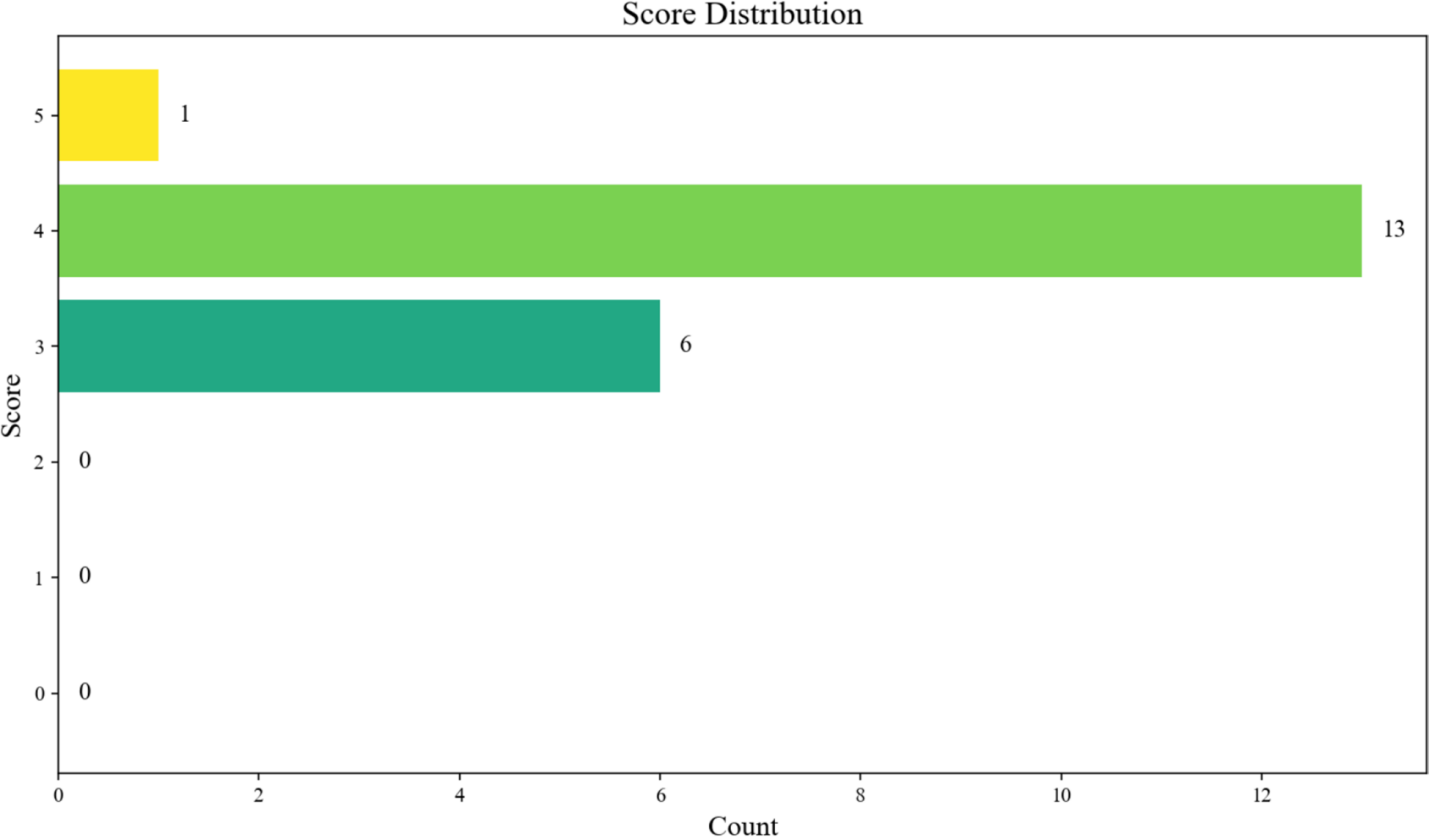
Expert Scoring Results

To strike a balance between getting experts involved and ensuring high completion rates, this study randomly selected 20 cases from the test set, including both predicted by KD + DPO and real results. Afterwards, the researchers turned these cases into a questionnaire for assessment. Two TCM experts participated in the assessment, both serving as Associate Professors and Associate Chief Physicians at the School of Traditional Chinese Medicine, Nanjing University of Chinese Medicine, and regularly practicing at Nanjing Municipal Hospital of Traditional Chinese Medicine. One expert was invited to score the outputs on a scale of 0–5 (evaluation time: 32 min), and the results are depicted in Fig. 18.

The overall average score was 3.75. Most ratings were 4, illustrating that the general assessment was moderately favorable and that experts viewed the model positively. Scores of 3 accounted for approximately 30% of the total, suggesting that while the model holds promise, there are still some shortcomings or areas that need enhancement. Aside from that, there were rare perfect cases scoring 5, mirroring that flawless performance was seldom achieved and further refinement is still indispensable.

**Fig. 19:**
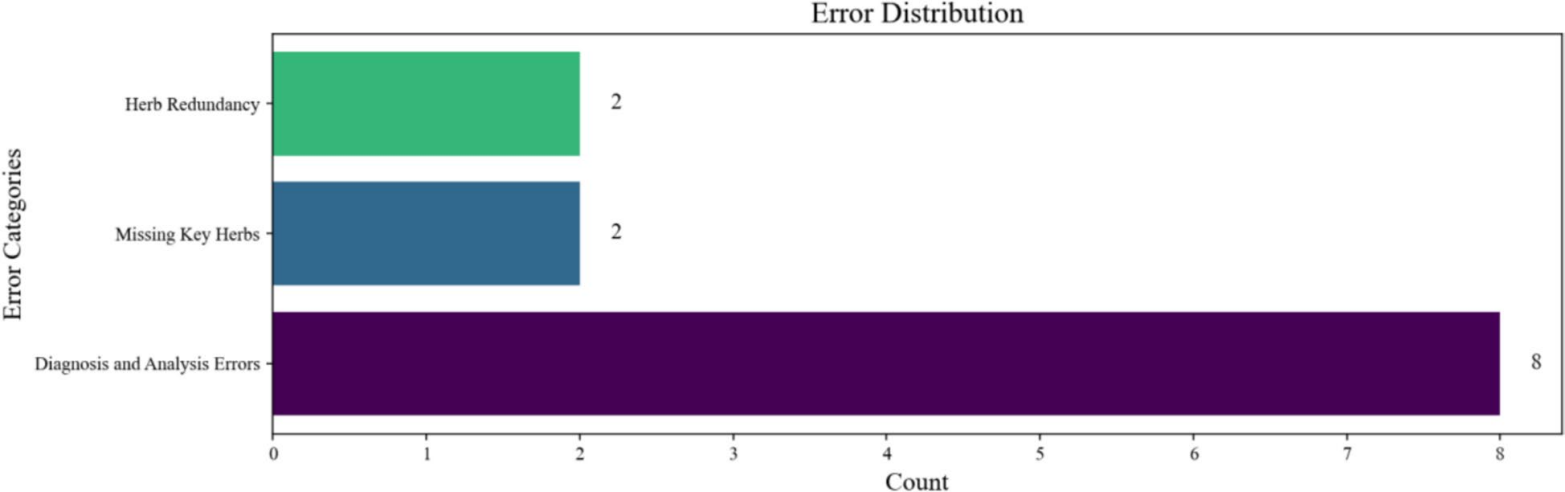
Expert Error Analysis Results

Simultaneously, another expert was invited to delve into the errors in the model outputs (evaluation time: 116 min), with error categories including Diagnosis and Analysis Errors, Missing Key Herbs, and Herb Redundancy. Multiple selections or no selection were permitted, and the distribution of results is revealed in Fig. 19.

As the above findings illustrate, the majority of errors were concentrated in Diagnosis and Analysis Errors. These issues predominantly stem from the fact that this study did not specifically optimize the diagnostic reasoning and analysis component of the model, and that the model still exhibits certain hallucination phenomena.

An error case is detailed as follows: abdominal distension, gastric pain with acid regurgitation, occasional soreness in the right lumbar-kidney region, dry mouth, spontaneous but slightly dry bowel movements, normal urination, tongue pale-yellow with thin greasy coating and dark-red body, pulse wiry and slippery. Model prediction is shown in Table 11.

**Table 11:** An error case

Description	Content
Real Prescription	pseudostellariae radix, glehniae radix, adenophorae, euphorbia helioscopia, hairyvein agrimonia, dwarf lilyturf root tuber, poria, coix seed, orange rind, barbated skullcup root, alismatis rhizoma, coptis, smilacis glabrae rhizoma, rersicae semen, parched massa medicata fermentata, processed chickens gizzard-membrane, rhizoma pinelliae preparatum, herba lycopi, euodiae fructus, polyporus, calcined arcae concha, bambusa tuldoides munro, sargassum, parched hedgehog skin, semen plantaginis, cyathulae radix
Ours: KD + DPO	*Patient Symptom Analysis* The patient suffered from abdominal distension, gastric pain with acid regurgitation, occasional soreness in the right lumbar-kidney region, dry mouth, spontaneous but slightly dry bowel movements, normal urination, tongue pale-yellow with thin greasy coating and dark-red body, pulse wiry and slippery. These signs imply a pattern of disharmony between the liver and stomach with concurrent liver–kidney yin deficiency. The disharmony between the liver and stomach accounts for the gastric distension, gastric pain, and acid regurgitation, while the soreness in the right lumbar and renal region suggests yin deficiency of the liver and kidney. The dry mouth and dry stools further reflect internal heat due to yin deficiency. *Recommended Prescription *radix paeoniae rubra, salvia miltiorrhiza, dandelion, radix bupleuri, curcumae radix, processed rhizoma cyperi, rhizoma pinelliae preparatum, orange rind, parched scutellaria radix, prunella spike, coptis, euodiae fructus, parched fructus aurantii, bambusa tuldoides munro, szechwan lovage rhizome, perilla stem, magnoliae officmalis flos, herba agastaches and perill *Prescription Explanation* 1. Soothe the Liver and Regulate Qi: radix bupleuri, curcumae radix, processed rhizoma cyperi, perilla stem, szechwan lovage rhizome. 2. Harmonize the Stomach and Relieve Pain: rhizoma pinelliae preparatum, orange rind, bambusa tuldoides munro, magnoliae officmalis flos. 3. Clear Heat and Drain Fire: parched scutellaria radix, prunella spike, coptis. 4. Invigorate Blood and Dispel Stasis: radix paeoniae rubra, salvia miltiorrhiza. 5. Clear the Liver and Benefit the Gallbladder: dandelion, parched fructus aurantii. 6. Tonify the Liver and Kidney: euodiae fructus. This prescription primarily focuses on soothing the liver, regulating qi, and harmonizing the stomach to relieve pain, supplemented with strategies to clear heat, invigorate blood, clear the liver and gallbladder, and tonify the liver and kidney, thereby targeting both the disharmony between the liver and stomach and the underlying yin deficiency of the liver and kidney

Experts put forth a viewpoint that the gastric distension, excess gastric acid and acid reflux should not be attributed to the liver; by contrast, they are more likely associated with the stomach itself. As demonstrated by the above phenomena, the model may have limitations in localizing the affected organ system, which in turn could lead to less favorable prescription recommendations. To cope well with this issue, future work will center around how to construct specialized evaluation models for diagnostic reasoning and analysis, coupled with preference modeling. In this way, the preference optimization can be applied to reduce both diagnostic errors and hallucinations. In addition, potential remedies may be considered, such as rule-based post-processing to correct inconsistent outputs and safety filters to prevent the generation of toxic or clinically forbidden herbs. Incorporating these strategies could enhance the safety and clinical reliability of the model in practical deployment.

## Conclusion

By proposing a two-stage training framework, this study aims to address multifarious key challenges in TCM prescription recommendation, namely limited explainability and the scarcity of top-quality preference data. This framework integrates knowledge distillation, achieved through conducting CoT supervised fine-tuning, along with implicit preference modeling via reinforcement learning guided by a lightweight model. The model not only maintains explainability and trustworthiness but also achieves notable enhancements in recommendation performance, which presents enormous potential for application in intelligent TCM-assisted diagnosis and treatment.

For example, in TCM education, this model could serve as an intelligent assistant to support physicians in learning and optimizing therapeutic strategies, with its partial explanation aiding comprehension. In outpatient practice, the model could be embedded into electronic medical record systems to provide real-time prescription recommendations and consistency checks, thereby achieving the reinforcement of diagnostic efficiency and medication safety. Nevertheless, what is noteworthy is that the developed LLM may still be constrained by the "hallucination" problem attributable to the relatively limited volume of training data. In other words, the developed LLM may generate outputs that deviate from authentic TCM knowledge or clinical common sense. Consequently, its practical application should always involve review and judgment by qualified TCM physicians to mitigate potential risks.

Nonetheless, quite a few drawbacks are still hanging around. First and foremost, the current training data are derived primarily from structured clinical records and have yet to fully incorporate diverse knowledge sources such as classical TCM literature, modern pharmacological research, and tongue image data, thereby giving rise to incomplete knowledge coverage. Aside from that, BART's pretraining corpus primarily originates from general-domain data, which constrains its capacity to capture the reasoning and analytical patterns inherent in clinical TCM case records, thereby limiting its explanation ability. On top of that, BART's preference outputs prioritize linguistic similarity over authentic clinical relevance, potentially falling short in reflecting real-world diagnostic performance or capturing TCM's holistic principles of syndrome differentiation and treatment. Consequently, despite reinforcements in surrogate evaluation indicators, this constrains its capacity to comprehensively capture authentic clinical preferences in real-world scenarios. Last but not least, the generated prescriptions haven't explicitly integrated the TCM formulation principle of "sovereign, minister, assistant, and courier", which suggests the designation of synergistic roles to herbs in a hierarchical structure, and it may weaken the explainability and systematic rigor of the generated prescriptions.

Future endeavors will center around the integration of multi-source knowledge, the expansion of preference signals, and the application of multi-modal learning. One direction involves the incorporation of TCM knowledge graphs, herb interaction principles, and canonical case corpora to enhance the model's knowledge completeness and logical coherence. Another viable option is to create multidimensional preference-modeling techniques that merge expert judgments, treatment efficiency, and safety elements, thereby boosting the clinical appropriateness of preference alignment. On top of that, by leveraging multi-modal data such as tongue and pulse images, it may be possible to advance the model's ability to reason across the Four Diagnostic Methods of TCM, "inspection, listening and smelling, inquiry, and palpation". In addition, expanding the dataset to encompass multiple institutions and leveraging multiple large language models for CoT generation will be essential for improving the generalizability and clinical robustness of the framework. Under such circumstances, it can lay a more robust foundation for building clinically practical and widely applicable intelligent diagnostic systems.

## Data Availability

The datasets used and/or analyzed in the current study are available from the corresponding author on reasonable request.

## Abbreviations

TCM: Traditional Chinese Medicine
LoRA: Low-Rank Adaptation
DPO: Direct Preference Optimization
BART: Bidirectional and Auto-Regressive Transformer
LLM: Large Language Model
CoT: Chain-of-Thought
TCMLLM-PR: Taditional Chinese Medicine Large Language Model for Prescription Recommendation
MACCTM: Multi-Agent Collaborative Chain-of-Thought Mechanism
DSRS: Dual-Stage Recovery Scheme
MLE: Maximum Likelihood Estimation
BERT: Bidirectional Encoder Representations from Transformers
GPT: Generative Pre-trained Transformer
BLEU: Bilingual Evaluation Understudy
ROUGE: Recall-Oriented Understudy for Gisting Evaluation
TFIDF: Term Frequency–Inverse Document Frequency
kNN: k-Nearest Neighbor
SimCSE: Simple Contrastive Sentence Embedding
KD: Knowledge Distillation
